# ProST: an image prompt-guided multimodal representation learning framework for spatial domain identification

**DOI:** 10.1093/bioinformatics/btag699

**Published:** 2026-09-22

**Authors:** Chenlan Sun, Zhengxia Wang, Qingchen Zhang, Xiaodong Bai, Chong Yin, Zhipei Sang, Hongsheng Xie, Yuxing Li

**Affiliations:** School of Computer Science and Technology, Hainan University, Haikou 570228, China; School of Computer Science and Technology, Hainan University, Haikou 570228, China; School of Computer Science and Technology, Hainan University, Haikou 570228, China; School of Computer Science and Technology, Hainan University, Haikou 570228, China; School of Computer Science and Technology, Hainan University, Haikou 570228, China; Key Laboratory of Tropical Biological Resources of Ministry of Education and Hainan Engineering Research Center for Drug Screening and Evaluation, School of Pharmaceutical Sciences, Hainan University, Haikou 570228, China; School of Computer Science and Technology, Hainan University, Haikou 570228, China; School of Computer Science and Technology, Hainan University, Haikou 570228, China

## Abstract

**Motivation:**

Spatial transcriptomics (ST) enables gene expression profiling while preserving the spatial organization of tissues, providing a powerful tool for dissecting tissue architecture and cellular heterogeneity. However, existing methods tend to prioritize improvements in clustering performance and overlook the fundamental objective of spatial domain identification, which is the recovery of spatial regions with coherent biological structures. As a result, the generated visualizations often fail to accurately capture fine-grained tissue organization. In addition, current approaches do not fully exploit the rich morphological and microenvironmental information embedded in histological images, which limits the representation learning capability in spatial domain identification.

**Results:**

We present *ProST*, an image *pro*mpt-guided multimodal representation learning framework for spatial domain identification on *ST* data. ProST is designed to effectively exploit morphology-aware information from histological images while reducing the impact of irrelevant visual noise, thereby generating robust and spatially coherent embeddings. We further optimized histological image feature extraction to improve the use of morphological information. Evaluations on multiple ST datasets demonstrate that ProST consistently outperforms existing methods in spatial domain identification, visualization, spatial trajectory inference, and gene expression imputation. Its high accuracy and strong generalization make ProST a powerful tool for resolving fine-grained tissue structures and revealing underlying biological complexity.

**Availability and implementation:**

ProST is implemented in Python and is freely available at https://github.com/Snake-Bio/ProST. The source code used in this study has been archived on Zenodo at DOI: 10.5281/zenodo.22144978. All datasets used in this study are publicly available at https://zenodo.org/records/22685502.

## 1 Introduction

The spatial organization of cells within tissues is essential for understanding biological function and disease-associated expression patterns. Spatial transcriptomics (ST) enables gene expression profiling while preserving spatial context, overcoming the limitations of conventional transcriptomic technologies and advancing the study of diverse biological systems (Longo *et al.* 2021).

Identifying spatial domains is a fundamental task in spatial transcriptomics, as it aims to partition tissue sections into spatially coherent regions according to gene expression patterns and spatial organization (Jin *et al.* 2024). Accurate domain identification is essential for understanding tissue architecture, cellular heterogeneity, and disease progression. Traditional clustering methods, such as k-means (Arthur and Vassilvitskii 2006) and Louvain (Blondel *et al.* 2008), mainly rely on transcriptomic similarity while largely ignoring spatial context, which limits their ability to recover biologically meaningful tissue structures.

Recent deep learning methods have substantially advanced spatial domain identification by modeling nonlinear relationships in high-dimensional ST data. STAGATE (Dong and Zhang 2022) employs a graph attention autoencoder to capture local spatial relationships and gene expression patterns, effectively producing spatially coherent embeddings. GraphST (Long *et al.* 2023) enhances spot representations via graph self-supervised contrastive learning, improving robustness in capturing spatial domain structure. SpaMask (Min *et al.* 2025) employs a dual masking graph autoencoder with contrastive learning to jointly model spatial and transcriptomic features, which strengthens the modeling of spot neighborhoods. STMGAMF (Fu *et al.* 2025) uses a multi-view graph convolutional network with adaptive adjacency and multi-strategy fusion to better represent complex spatial patterns. Despite their success, these methods underutilize the rich morphological information in histological images, limiting full multimodal exploitation in spatial transcriptomics.

To address this limitation, several studies have incorporated histological images into spatial clustering. DeepST (Xu *et al.* 2022) extracts image features with a convolutional backbone, but mainly uses them to refine spatial adjacency rather than directly guide transcriptomic representation learning. SpaICL (Zhao and Min 2025) further integrates histological image features with gene expression and spatial information through graph contrastive learning, dual cross-attention, and curriculum learning, enabling deeper multimodal fusion.

However, most existing methods primarily focus on improving clustering performance through multi-view contrastive learning or graph neural network architectures, while often overlooking the fundamental goal of spatial domain identification, namely the recovery of spatially coherent biological regions. As a result, the biological interpretability of learned representations is frequently sacrificed for performance-oriented optimization. Although these methods may achieve competitive quantitative scores, their visualized spatial domains often exhibit over-smoothing effects, making it difficult to capture key biologically meaningful structures such as variations in cortical layer thickness, boundary consistency across regions, and intra-layer morphological stability. Furthermore, histopathological images contain rich morphological and microenvironmental information highly relevant to tissue organization, but these signals are often underutilized in existing frameworks. This is mainly because most methods either treat image features as auxiliary inputs or directly fuse them with gene expression, without explicitly distinguishing their fundamentally different reliability and semantic properties. In practice, histopathological images are inherently noisy and heterogeneous, suffering from staining variations, tissue sectioning artifacts, and scanner-induced distortions, which significantly limit their direct applicability in representation learning. Therefore, a key challenge lies in effectively exploiting biologically meaningful morphological information while suppressing irrelevant visual perturbations.

To overcome these issues, we propose ProST, a novel pathology-image prompt-guided gene representation learning framework for spatial domain identification (Fig. 1). Inspired by the prompting mechanism of LLMs, ProST uses histological images as morphology-aware prompts to guide gene-centered representation learning, rather than directly fusing image features with gene expression. It adopts a progressive design consisting of three coordinated components: the Image Prompt Pool (IPP), which compresses histopathological image features into compact representations capturing microenvironmental patterns and shared tissue structures; the Spatially Constrained Asymmetric Transformer (SCAT), which injects prompt information into gene representations under biologically meaningful constraints to prevent uncontrolled information propagation; and the Gene-centered Adaptive Fusion (GAF), which adaptively integrates multimodal information while preserving gene expression as the dominant representation of biological state. ProST is further trained under a graph masked autoencoder and contrastive self-supervised learning framework, with an optimized scaled cosine error (SCE) loss to improve robustness against heavy masking, noise, and sparsity in spatial transcriptomics data. In addition, we optimize the image feature extraction pipeline by systematically evaluating multiple patch cropping strategies and histological feature extractors, aiming to fully extract informative microenvironmental features while reducing interference from neighboring spots.

**Figure 1:**
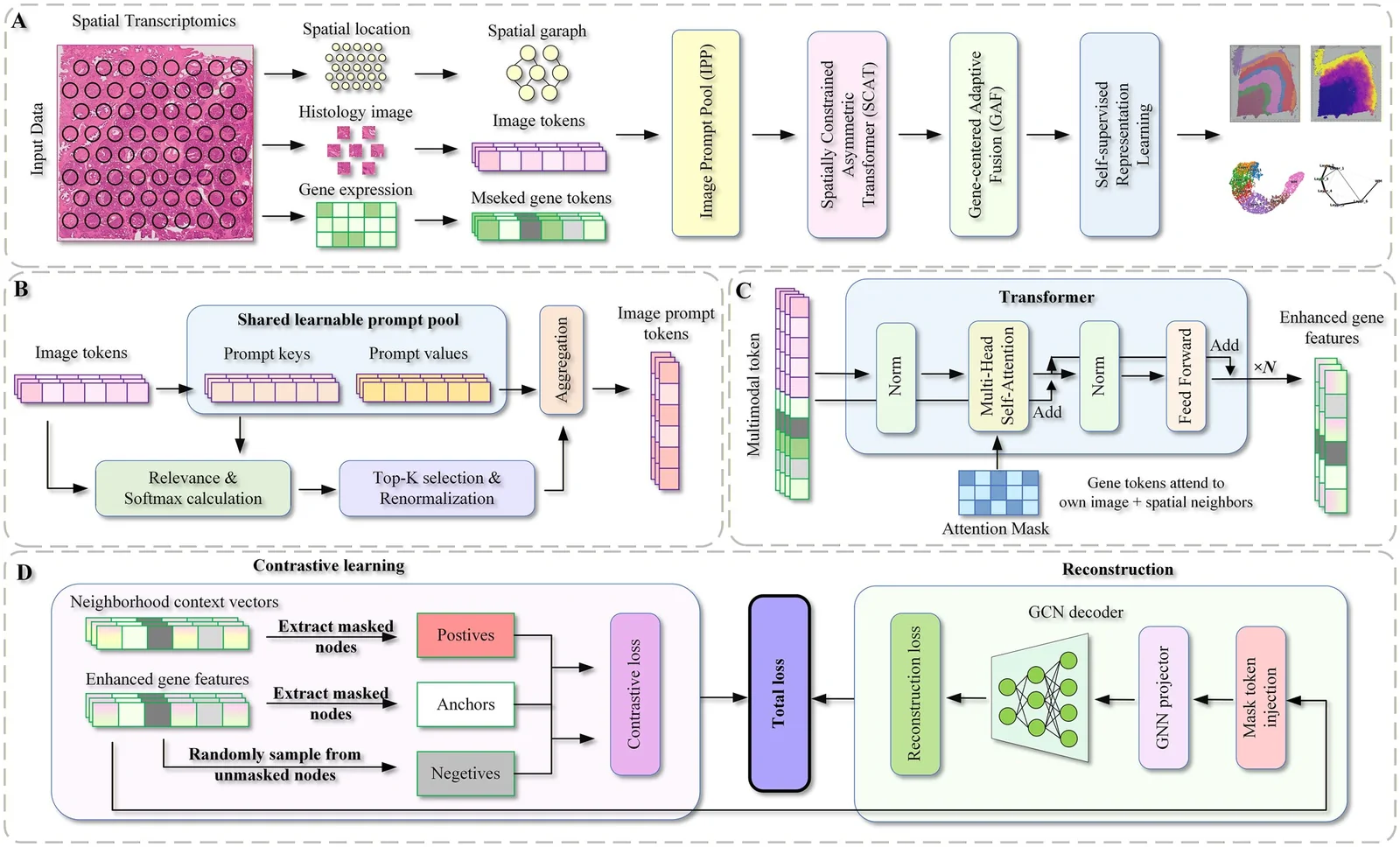
(A) Overview of ProST. (B) Image Prompt Pool (IPP). (C) Spatially Constrained Asymmetric Transformer (SCAT). (D) Self-supervised representation learning.

Through the joint optimization of reconstruction and contrastive objectives, ProST learns robust, spatially coherent, and biologically informative gene-centered embeddings. The learned embeddings are subsequently clustered using mclust (Scrucca *et al.* 2016) for spatial domain identification. These embeddings further support multiple downstream analyses, including visualization, spatial trajectory inference, and gene expression imputation. Experimental results across multiple spatial transcriptomics datasets demonstrate that ProST not only outperforms six widely used methods in quantitative evaluation metrics, but also exhibits superior qualitative performance in visualization results, producing spatial domains that are more consistent with underlying biological structures and showing stronger generalization ability.

## 2 Materials and methods

### 2.1 Data preprocessing

Given an ST dataset with *N* spatial spots, ProST uses three types of input information: gene expression profiles, spatial coordinates, and histological image patches.

For transcriptomic preprocessing, we use SCANPY (Wolf *et al.* 2018) to remove genes and spots without detectable expression, log-normalize the remaining matrix, select the top 2000 highly variable genes, and apply principal component analysis to obtain compact gene features $X_{\text{PCA}}\in R^{N\times N_{\text{PCA}}}$.

For histological image preprocessing, an image patch is cropped for each spot from the matched whole-slide image. We systematically evaluated four patch cropping strategies based on the spot diameter *D* and the nearest-neighbor spot center distance *d*: (1) patch size equal to *D*, covering only the spot itself; (2) patch side length equal to *d*, producing non-overlapping local patches; (3) maximum side length 2d−D, avoiding neighboring spot centers while allowing moderate contextual overlap; and (4) patch side length 2*d*, capturing a broader tissue context. In the default setting of ProST, we use the spot-diameter-based strategy, which focuses on spot-specific morphology while reducing interference from neighboring spots. The comparison of different cropping strategies is reported in Supplementary Text 3 and Fig. S1, available as supplementary data at *Bioinformatics* online.

All patches are resized to 224×224 pixels and encoded using the Universal Histopathology Image Encoder (UNI) (Chen *et al.* 2024), which was selected as the most effective encoder based on multiple comparative experiments in our study, yielding the image feature matrix $F\in R^{N\times 1024}$ (Fig. S2, available as supplementary data at *Bioinformatics* online).

To incorporate spatial location information, we construct a *k*-nearest-neighbor (KNN) graph from spot coordinates, resulting in an undirected graph G=(V,E) with adjacency matrix A. This graph serves as a spatial prior for downstream modules, including neighborhood context computation for contrastive learning and graph-based projection and decoding in the masked gene reconstruction module.

### 2.2 Multimodal token construction

To support self-supervised learning, ProST randomly masks a subset of spots with ratio ρ (default ρ=0.6, details in Supplementary Text 4, available as supplementary data at *Bioinformatics* online). Gene and image features are then projected into a shared latent space. For each spot *i*, the masked gene feature ${\tilde{x}}_{i}$, corresponding to the *i*-th row of $\tilde{X}$, is mapped to a gene token $g_{i}$:


(1) $$g_{i}=W_{g}{\tilde{x}}_{i},$$

where $W_{g}$ is a learnable gene projection matrix.

Similarly, the image feature $f_{i}$, corresponding to the *i*-th row of the image feature matrix F, is mapped to an image token $h_{i}$:


(2) $$h_{i}=W_{f}f_{i},$$

where $W_{f}$ is a learnable image projection matrix.

To adapt general histopathology features to ST representation learning, ProST further refines the image token through a lightweight image adapter:


(3) $${\tilde{h}}_{i}=h_{i}+\psi (h_{i}),$$

where ψ(·) denotes a small feed-forward network. The refined image token ${\tilde{h}}_{i}$ is then used by IPP to retrieve morphology-aware prompts.

### 2.3 Image Prompt Pool (IPP)

Histological image features provide rich morphological context for spatial domain identification, but they are often affected by staining variation, scanning noise, and sectioning artifacts. Directly propagating these raw visual representations into multimodal fusion may introduce instability and reduce the reliability of downstream transcriptomic representation learning. To address this issue, we propose an Image Prompt Pool (IPP), which serves as a morphology-aware memory bank that stores a set of learnable prototype prompts. Specifically, IPP compresses histopathological image features into compact representations that effectively capture microenvironmental patterns and shared tissue structures across spots. Instead of directly using raw image tokens, IPP retrieves these compact prompt representations, providing stable morphological information that reflects microenvironments and shared structures, which guides multimodal fusion and improves transcriptomic learning.

Formally, the prompt pool is defined as a set of learnable key-value pairs:


(4) $$P={\left\{(k_{m},u_{m})\right\}}_{m=1}^{M},$$

where $k_{m}\in R^{d}$ represents the *m*-th prototype key that encodes a morphological cluster center, and $u_{m}\in R^{d}$ denotes its corresponding prompt embedding.

For each spatial spot *i*, the refined image token ${\tilde{h}}_{i}$ is used as a query to retrieve relevant morphological prototypes. The similarity between the image token and each prompt key is computed as:


(5) $$s_{im}=\frac{{\tilde{h}}_{i}^{⊤}k_{m}}{\tau_{p}},$$

where $\tau_{p}$ is a temperature parameter that controls the sharpness of retrieval.

To ensure sparse and noise-resistant retrieval, we first compute a soft assignment over all prompts:


(6) $$a_{im}=\frac{\;\text{exp}(s_{im})}{\sum_{j=1}^{M}\;\text{exp}\;(s_{ij})}.$$

Then, instead of using all prompts, we apply a sparsification operator that retains only the top-*K* most relevant prompts:


(7) $$T_{i}=\mathrm{TopK}(a_{i1},\ldots ,a_{iM}),$$

where $T_{i}$ denotes the selected index set for spot *i*.

The retained weights are re-normalized over the selected subset:


(8) $${\hat{a}}_{im}=\frac{a_{im}}{\sum_{j\in T_{i}}a_{ij}},\;m\in T_{i}.$$

Finally, the morphology-aware image prompt for each spot is constructed via a sparse weighted aggregation:


(9) $$p_{i}=\sum_{m\in T_{i}}{\hat{a}}_{im}u_{m}.$$

This design enables IPP to function as a *morphology prototype memory*, where each spot is represented as a sparse combination of shared histological prototypes. As a result, visually similar spots tend to retrieve similar prompt compositions, while irrelevant or noisy visual patterns are effectively suppressed. The resulting image prompt $p_{i}$ is then used to guide gene-centered representation learning in the SCAT module.

### 2.4 Spatially Constrained Asymmetric Transformer (SCAT)

After obtaining the morphology-aware image prompt $p_{i}$ from IPP and the gene token $g_{i}$ from masked gene modeling, ProST injects the image prompt into the corresponding gene token through an additive prompt-guidance mechanism:


(10) $$g_{i}^{\prime }=g_{i}+\alpha p_{i},$$

where α is a learnable scaling parameter that controls the contribution of the retrieved image prompt. This additive design allows histological morphology to guide gene representation learning without treating image-derived prompts as an independent information source equivalent to gene expression.

ProST then constructs a multimodal token sequence by concatenating the refined image tokens and the prompt-enhanced gene tokens:


(11) $$T^{\left(0\right)}=[{\tilde{h}}_{1},\ldots ,{\tilde{h}}_{N},g_{1}^{\prime },\ldots ,g_{N}^{\prime }],$$

where the first *N* tokens correspond to image tokens and the remaining *N* tokens correspond to prompt-enhanced gene tokens. Each gene token therefore carries both its original transcriptomic representation and morphology-aware guidance derived from histological images.

Unlike conventional Transformer architectures that allow unrestricted token interactions, SCAT introduces a structure-aware attention mechanism that constrains information flow via a predefined binary attention mask. Instead of modifying the Transformer architecture, we retain the standard Transformer formulation and enforce biological constraints through the attention mask, ensuring that only meaningful interactions are permitted between modalities and spatial neighborhoods.

The attention operation in each Transformer layer is defined as:


(12) $$\text{Attention}(Q,K,V)=\text{softmax}\left(\frac{QK^{⊤}}{\sqrt{d_{k}}}+M\right)V,$$

where *Q*, *K*, and *V* are the query, key, and value matrices, $d_{k}$ is the dimension of the key vectors, and $M\in {\left\{0,¯\infty \right\}}^{2N\times 2N}$ is a binary attention mask that restricts invalid interactions by assigning ¯∞ to masked positions.

The asymmetric attention mask permits only three types of interactions: self-attention for all tokens ($M_{i,i}=0$ for i=1,…,2N), each gene token attending to its paired image token ($M_{N+i,i}=0$ for i=1,…,N), and gene tokens of spatially neighboring spots attending to each other ($M_{N+i,N+j}=0$ for every edge (i,j)∈E). All other entries of *M* are set to ¯∞, preventing image tokens from attending to any other token and gene tokens from attending to non-adjacent gene tokens or non-paired image tokens. This enforces the asymmetric, spatially constrained information flow of SCAT.

After *L* Transformer layers, the output token sequence is:


(13) $$T^{\left(L\right)}=[{\hat{z}}_{1}^{I},\ldots ,{\hat{z}}_{N}^{I},{\hat{z}}_{1}^{G},\ldots ,{\hat{z}}_{N}^{G}],$$

where ${\hat{z}}_{i}^{I}$ denotes the image-side representation and ${\hat{z}}_{i}^{G}$ denotes the prompt-guided gene-side representation of the *i*-th spot. These representations are subsequently fed into the gene-centered adaptive fusion module for final embedding construction.

### 2.5 Gene-centered Adaptive Fusion (GAF)

After the SCAT module produces the image-side representations ${\hat{z}}_{i}^{I}$ and prompt-guided gene-side representations ${\hat{z}}_{i}^{G}$ for each spot, ProST introduces a Gene-centered Adaptive Fusion (GAF) module to obtain the final spot embedding. The key idea of GAF is to model multimodal integration as a reliability-aware correction process over a gene-centered representation space. Gene expression is treated as the primary observation that directly reflects cellular state, while histological morphology provides auxiliary contextual evidence that may be noisy but informative.

From a probabilistic perspective, we interpret the gene-side representation ${\hat{z}}_{i}^{G}$ as a stable estimate of the latent biological state, while the image-side representation ${\hat{z}}_{i}^{I}$ acts as an auxiliary observation that provides an uncertain correction signal. The role of fusion is therefore to adaptively control how much the auxiliary modality should correct the gene-based estimate.

We define a reliability function over image-derived representations:


(14) $$\lambda_{i}=\sigma (f_{\lambda }({\hat{z}}_{i}^{I})),$$

where $f_{\lambda }(\cdot )$ maps morphological features into a reliability score, and σ(·) normalizes it into (0,1), which can be interpreted as the confidence of image-derived correction.

The final embedding is formulated as a reliability-weighted residual update:


(15) $$z_{i}={\hat{z}}_{i}^{G}+\eta \lambda_{i}{\hat{z}}_{i}^{I},$$

where η controls the global strength of cross-modal correction.

This formulation is a probabilistic refinement: gene expression sets the base estimate of the latent domain, while morphology provides a confidence-weighted correction. The gating value rises with histological reliability, applying stronger correction, and falls when visual signals are noisy or ambiguous, suppressing image input. Unlike symmetric fusion, it explicitly models modality imbalance and uncertainty, keeping gene expression dominant and morphology as an adaptive, reliability-dependent corrective signal.

### 2.6 Self-supervised representation learning

After GAF produces the final spot embedding $z_{i}$, ProST is trained under a self-supervised framework combining spatial contrastive learning and an optimized scaled cosine error (SCE) reconstruction, ensuring robustness against heavy masking, noise, and sparsity.

For spatial contrastive learning, a local context vector is computed for each spot *i* by averaging its spatial neighbors:


(16) $$c_{i}=\sigma (\frac{1}{\left|N(i)\right|}\sum_{j\in N(i)}z_{j}),$$

where N(i) is defined by the spatial graph and σ is the sigmoid function. Each masked spot embedding $z_{i}$ serves as the anchor, its local context $c_{i}$ as the positive, and a randomly sampled embedding as the negative. Cosine similarities are


(17) $$s_{i}^{+}=\frac{z_{i}^{⊤}c_{i}}{∥z_{i}∥∥c_{i}∥},\;s_{i}^{-}=\frac{z_{i}^{⊤}z_{r(i)}}{∥z_{i}∥∥z_{r(i)}∥},$$

and the spatial contrastive loss is


(18) $$L_{\mathrm{con}}=-\frac{1}{\left|M\right|}\sum_{i\in M}\;\text{log}\;\frac{\;\text{exp}(s_{i}^{+}/\tau )}{\;\text{exp}(s_{i}^{+}/\tau )+\text{exp}(s_{i}^{-}/\tau )}.$$

For masked gene reconstruction, embeddings of masked spots are replaced with a learnable vector $m_{r}$ and decoded via graph-based projection and decoder:


(19) $$H=W_{d}Z,\;\hat{X}=f_{\mathrm{dec}}(f_{\mathrm{proj}}(H,G),G).$$

The optimized SCE loss is defined as


(20) $$L_{\mathrm{rec}}=\frac{1}{\left|M\right|}\sum_{i\in M}{\left[-\text{log}\;\frac{1+\text{cos}({\hat{x}}_{i},x_{i})}{2}\right]}^{\gamma }.$$

This SCE emphasizes directional consistency of gene expression vectors rather than exact magnitudes, making it more robust than MSE to sparsity, scale variation, and noise. While originally proposed in GraphMAE (Hou *et al.* 2022), our optimized version further adaptively emphasizes difficult-to-reconstruct spots and integrates graph-based projection to leverage neighborhood context, producing spatially coherent and biologically informative embeddings.

The total self-supervised loss is


(21) $$L=\lambda_{\mathrm{rec}}L_{\mathrm{rec}}+\lambda_{\mathrm{con}}L_{\mathrm{con}}.$$

## 3 Results

### 3.1 Datasets and evaluation metrics

We evaluate ProST on five publicly available spatial transcriptomics datasets with matched histological images: human dorsolateral prefrontal cortex (DLPFC) (Maynard *et al.* 2021), human breast cancer (HBC) (Polyak 2011), mouse brain tissue sections including anterior sagittal (MBA), posterior sagittal (MBP), and coronal (MBC) (Sunkin *et al.* 2013), HER2-positive breast tumors (HER2+) (Andersson *et al.* 2021), and chicken heart sections at developmental days 7, 10, and 14 (Mantri *et al.* 2021); these datasets are described in detail in Supplementary Text 1, available as supplementary data at *Bioinformatics* online. ProST is compared with six widely used deep learning-based methods, including STMGAMF, SpaICL, SpaMask, GraphST, DeepST, and STAGATE. Evaluation metrics, including Adjusted Rand Index (ARI) and Normalized Mutual Information (NMI), are described in detail in Supplementary Text 2, available as supplementary data at *Bioinformatics* online.

### 3.2 Results on the DLPFC dataset

The results demonstrate that ProST achieves the highest clustering performance across all 12 tissue slices (Fig. 2B). Slice 151675 characterized by subtle inter-layer differences, provides a representative case for evaluating precision. Visualization shows that ProST accurately delineates cortical layers, identifying even the thinnest layers (1, 2, and 4) while preserving their relative thickness, whereas several baseline methods either fail to detect these thin layers or incorrectly expand them into thicker regions (Fig. 2C). The identified spatial domains closely align with manual annotations, particularly at boundaries where many baselines exhibit domain mixing. These comparisons indicate that ProST effectively leverages histological image information to recover fine-grained tissue architecture, thereby enabling more accurate spatial domain identification.

**Figure 2:**
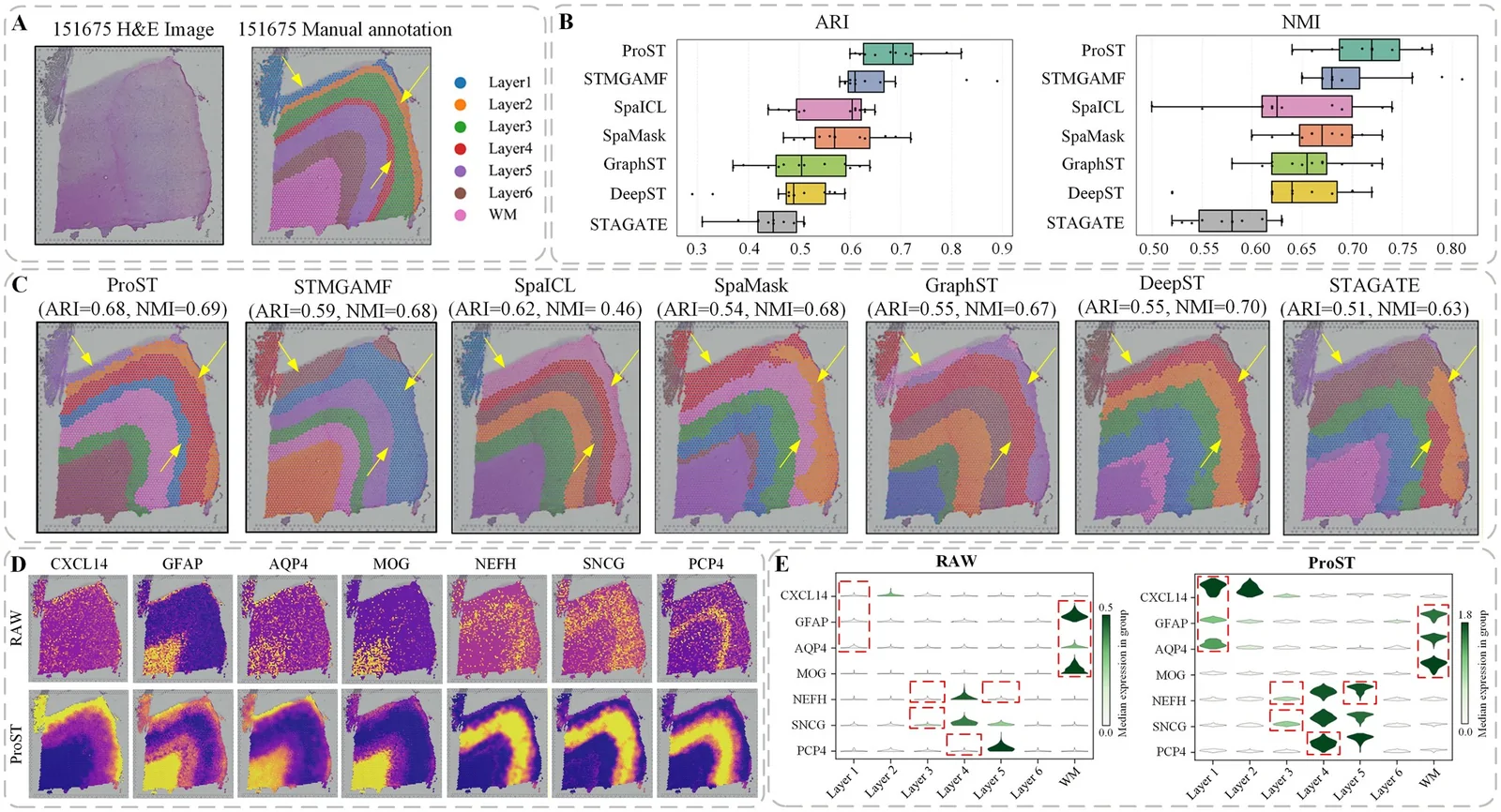
Results on the DLPFC dataset. (A) Manual annotation of cortical layers in slice 151675. (B) ARI and NMI comparison across 12 DLPFC slices. (C) Spatial domain identification results on slice 151675. (D) Spatial patterns of representative layer-associated marker genes. (E) Violin plots of marker gene expression across cortical layers.

We further examined whether ProST can recover biologically meaningful spatial expression patterns by analyzing representative layer-associated marker genes (Fig. 2D). In slice 151675 ProST-enhanced representations revealed clearer laminar enrichment patterns than the raw data, which were affected by noise, sparsity, and dropout. For example, ProST strengthened the layer 1 enrichment of the weak and diffuse *CXCL14* signal and clearly enriched the white-matter marker *MOG* in the white matter region. For genes with broader or noisier expression, ProST-based denoising and imputation revealed enrichment in both white matter and layer 1 for *GFAP*, consistent with astrocyte and glial/vascular cell distributions in the human DLPFC, and *AQP4* similarly showed a clearer layer 1 and white-matter pattern after ProST processing, in line with previous reports (Maynard *et al.* 2021). For neuronal layer-associated genes, ProST refined spatial patterns: *NEFH* and *SNCG* were upregulated in layer 3, and *PCP4* was enriched in layer 5. Overall, ProST not only reconstructs classical layer markers but also extracts anatomically meaningful spatial enrichment from genes with low signal-to-noise ratios, sparse expression, or broad distributions, enhancing both neuronal layer-specific and non-neuronal microenvironment-associated signals. The violin plots further show that ProST markedly improves spatial resolution and signal intensity of layer marker genes compared with raw data (Fig. 2E), demonstrating its effectiveness in spatial denoising and enhancing layer-associated expression patterns.

Overall, these results highlight the robustness of ProST in resolving fine-grained spatial domains and capturing biologically coherent patterns across the cortex. Detailed comparisons of all methods across the 12 DLPFC slices are provided in Fig. S3, available as supplementary data at *Bioinformatics* online.

### 3.3 Results on the HBC dataset

We evaluated ProST on the HBC dataset to assess its ability to resolve complex tumor microenvironments. Unlike DLPFC, this dataset exhibits highly heterogeneous tissue structures and intricate microenvironmental organization. The tissue was manually annotated by a pathologist into four major morphotypes, including ductal carcinoma in situ/lobular carcinoma in situ (DCIS/LCIS), healthy tissue, invasive ductal carcinoma (IDC), and tumor-edge regions, and further subdivided into 20 subregions (Fig. 3A).

**Figure 3:**
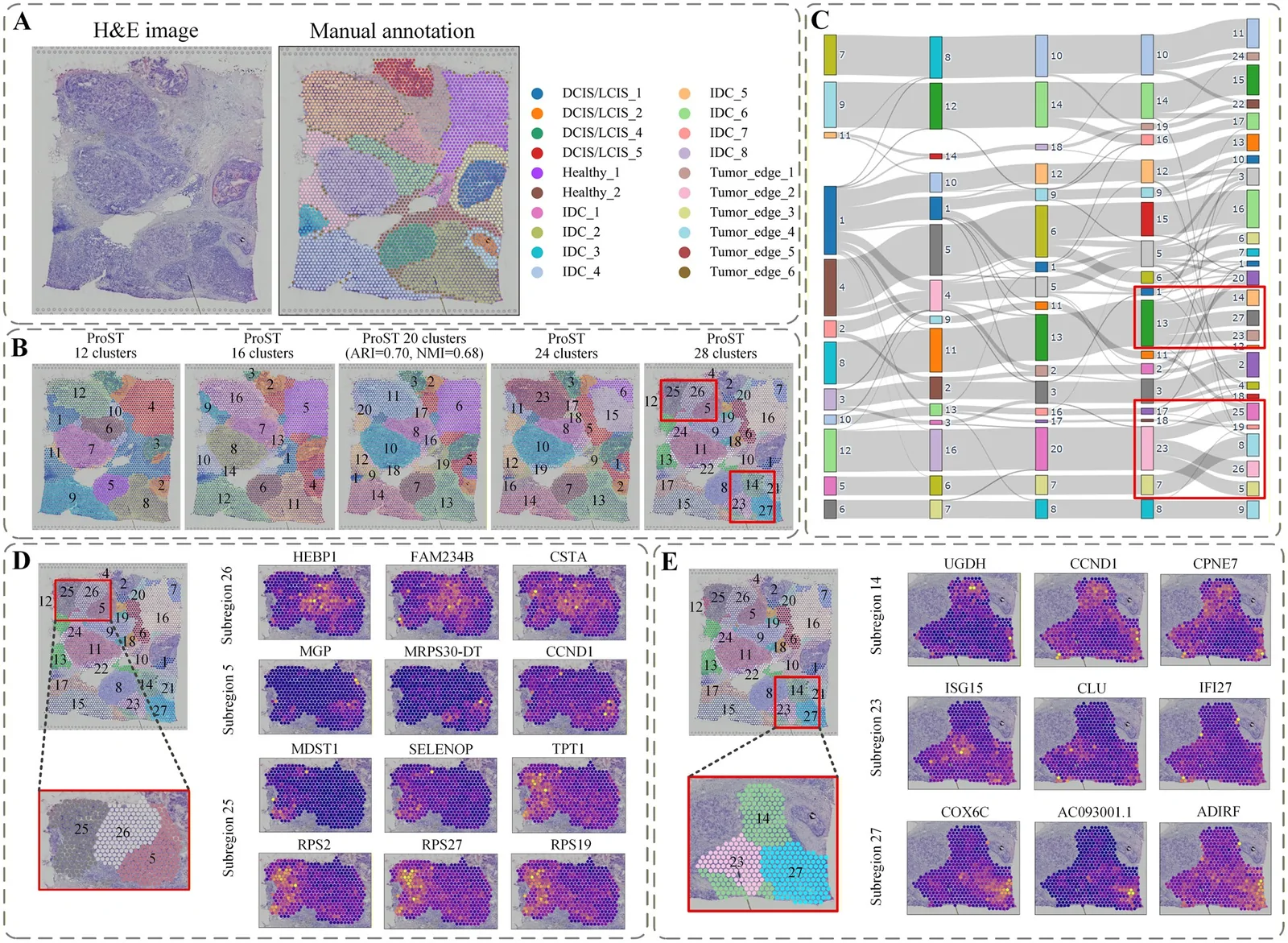
Results on the HBC dataset. (A) H&E image and manual annotation of human breast cancer tissue. (B) Spatial domain detection results of ProST with different cluster numbers. (C) Sankey diagram comparing the spatial domains generated by ProST with different cluster numbers. The widths of the lines linking two sets of clusters correspond to the number of spots they have in common. Due to the randomness inherent in the mclust clustering algorithm, if the intersection of spots between the source and target domains is less than 5, the target domains are filtered out in the corresponding layer of the Sankey diagram. (D) Visualization of domains 25, 26, and 5 when cluster=28, together with the expression of their representative marker genes. (E) Visualization of domains 14, 23, and 27 when cluster=28.

To evaluate spatial domain identification at different clustering resolutions, we applied ProST with cluster numbers of 12, 16, 20, 24, and 28 (Fig. 3B). At coarse resolutions, ProST distinguished major pathological compartments, while increasing cluster numbers revealed finer intratumoral substructures within IDC regions. The Sankey diagram illustrates that broad tumor-associated regions were progressively decomposed into more fine-grained subdomains as the cluster number increased, preserving continuity between coarse and fine spatial partitions (Fig. 3C). When the cluster number was set to 28, ProST identified domains 25, 26, and 5 within the IDC_5 region, and domains 14, 23, and 27 within the IDC_2 region, indicating that morphologically similar IDC regions could be further decomposed into molecularly distinct subregions, reflecting substantial intratumoral heterogeneity not fully captured by coarse manual annotations.

Differential expression analysis using the Wilcoxon test revealed domain-specific gene enrichment patterns (Fig. S6, available as supplementary data at *Bioinformatics* online). In IDC_5, domain 26 was enriched for *CSTA*, *FAM234B*, and *HEBP1*, suggesting an association with tumor progression, consistent with previous reports (Li *et al.* 2024). Domain 5 showed enrichment of *MGP*, *MRPS30-DT*, and *CCND1*, indicative of proliferative activity and microenvironment remodeling. Domain 25 exhibited high expression of *MDST1*, *SELENOP*, *TPT1*, and ribosomal genes (*RPS2*, *RPS27*, *RPS19*), reflecting enhanced translational and metabolic activity.

In IDC_2, domain 14 was enriched for *UGDH*, *CCND1*, and *CPNE7*, indicating proliferative and extracellular matrix remodeling characteristics; domain 23 showed enrichment for *ISG15*, *CLU*, and *IFI27*, reflecting immune activation and inflammation-associated features; and domain 27 was enriched for *COX6C*, *AC093001.1*, and *ADIRF*, suggesting metabolic and mitochondrial activity adaptation.

Taken together, these domain-specific gene enrichment patterns reveal substantial molecular heterogeneity within histologically similar IDC regions. These results indicate that ProST provides an effective framework for resolving spatially organized tumor heterogeneity and may facilitate the discovery of biologically meaningful tumor substructures relevant to disease progression and therapeutic response. Detailed comparisons with other methods on the HBC dataset are provided in Fig. S5, available as supplementary data at *Bioinformatics* online.

### 3.4 Results on the mouse brain, HER2+, and chicken heart datasets

We further evaluated ProST on the Mouse Brain Anterior (MBA) dataset, which contains complex and fine-grained anatomical structures. ProST achieved the best clustering performance among all compared methods, with an ARI of 0.60 and an NMI of 0.74, the first method to exceed an ARI of 0.60 on this dataset. Spatial visualization shows that ProST recovers both major anatomical regions and finer subdivisions, particularly in structurally complex areas such as the olfactory bulb and cortical regions. For example, MOB::Gr and MOB::Opl correspond to the granule cell layer and outer plexiform layer of the main olfactory bulb, while MO::L5 and SS::L5 represent layer 5 of motor and somatosensory areas. Baseline methods often produced overly smoothed transitions and incorrectly merged distinct biological structures into continuous thin layers, whereas ProST accurately delineated these fine-grained regions, demonstrating its strength in resolving complex brain architecture.

For the HER2+ dataset, which contains highly heterogeneous breast tumor tissue, ProST was applied to all eight slices. Figure 4C shows that ProST consistently outperformed the six other methods in both ARI and NMI across slices. Visualization of the identified spatial domains (Fig. 4D) indicates that ProST more accurately delineates tumor, connective tissue, and adipose tissue regions, with higher regional continuity and reduced fragmentation. In particular, ProST precisely identified the invasive cancer region in slice B1 and approximately delineated the connective tissue region, benefiting from the morphological heterogeneity present in the H&E images.

**Figure 4:**
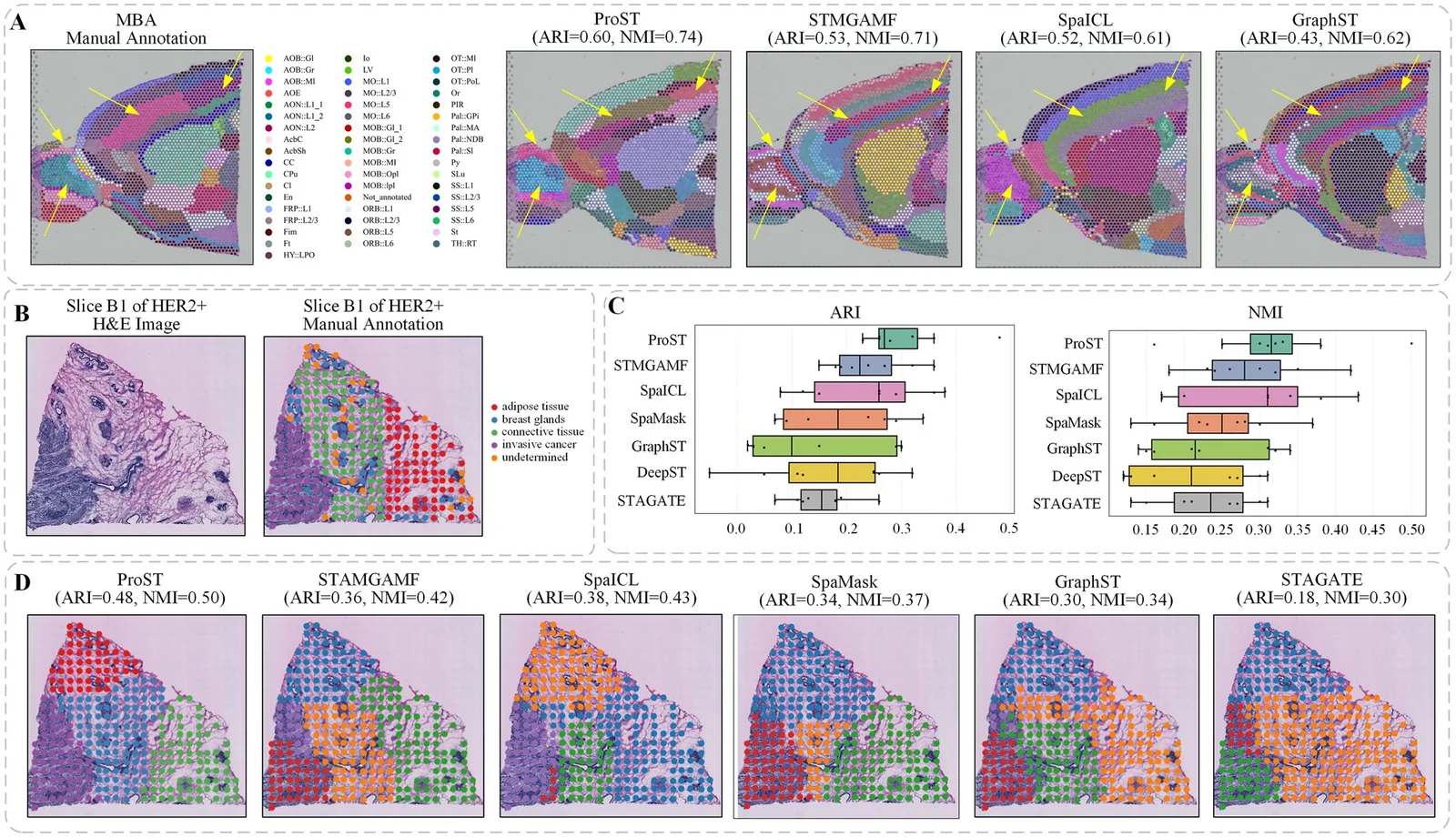
(A) Spatial domain identification and clustering performance comparison across methods for the MBA dataset. (B) Slice B1 of the HER2+ dataset showing manual annotation of tissue regions. (C) ARI and NMI comparison across 8 HER2+ slices with six other methods. (D) Comparison of spatial domain identification results in the HER2+ dataset.

For the mouse brain posterior sagittal (MBP) and coronal (MBC) sections, ProST also achieved strong performance (Supplementary Figs. S9 and S10, available as supplementary data at *Bioinformatics* online). On the chicken heart datasets at developmental days 7, 10, and 14, ProST outperformed all other compared methods, demonstrating its generalizability across non-mammalian cardiac tissues (Supplementary Figs S11–S13, available as supplementary data at *Bioinformatics* online).

### 3.5 Ablation studies

We conducted ablation studies on DLPFC, HBC, and MBA to evaluate key design choices in ProST. The full model was compared with seven variants, including No-IPP, SCAT-Unmasked, SCAT-CrossAttention, SCAT-MaskedCrossAttention, No-GAF, SCE-Original, and MSE (Fig. 5). Removing IPP reduced the benefit of morphology-aware prompts, confirming that pathology images provide complementary information beyond gene expression and spatial coordinates. Modifying SCAT, either by removing spatial constraints or replacing it with conventional cross-attention, impaired spatially constrained multimodal interaction, indicating that unrestricted attention is insufficient for spatial transcriptomics data. The performance drop of No-GAF demonstrated that gene-centered adaptive fusion is necessary to balance molecular and visual cues, and the reconstruction-loss variants further validated the optimized SCE objective. Overall, these results confirm the complementary roles of IPP, SCAT, GAF, and the SCE loss in integrating multimodal information for accurate spatial domain identification.

**Figure 5:**
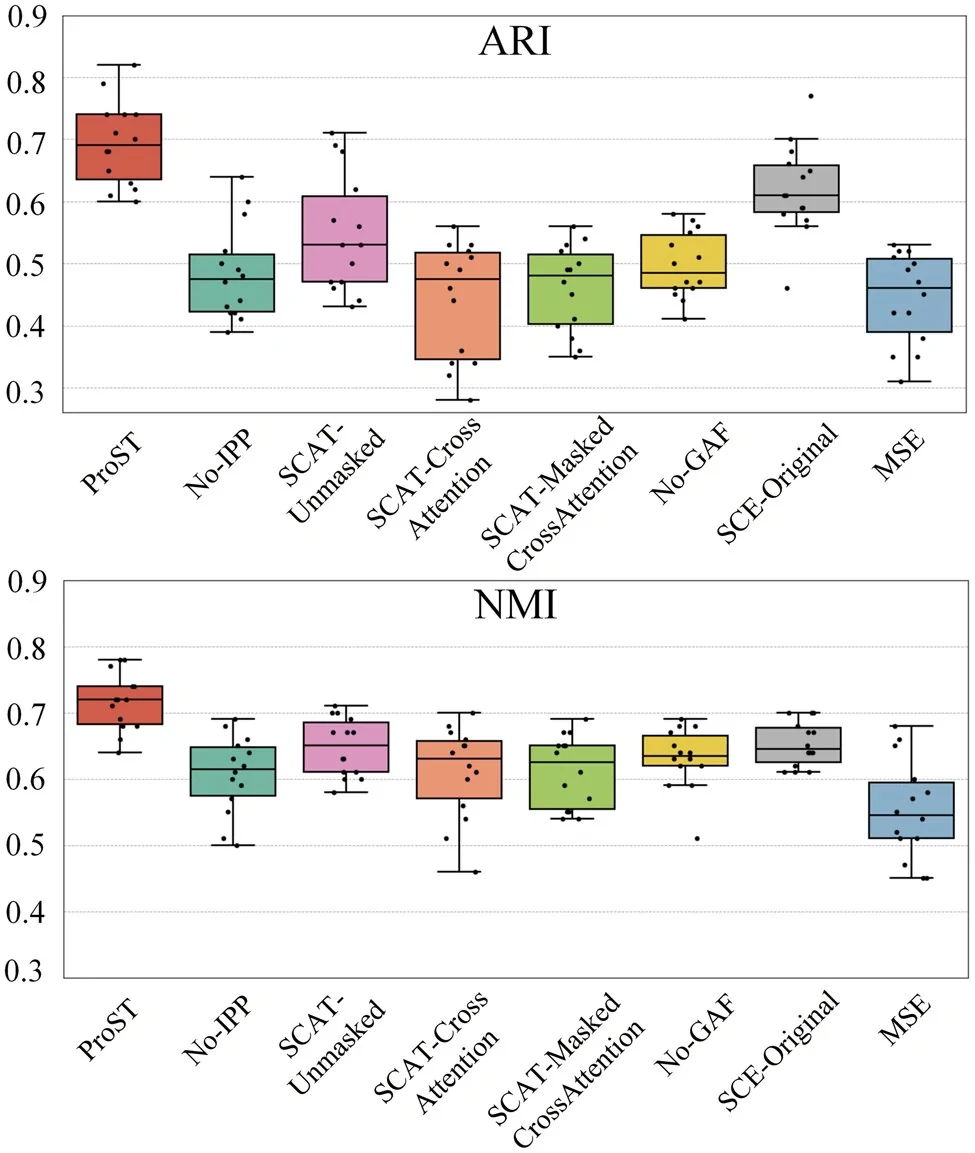
Boxplots showing the ablation study results of ProST.

## 4 Discussion

ProST is motivated by three key challenges in existing methods: sparse and noisy gene expression, insufficient recovery of biologically coherent spatial domains, and underutilization of morphological information in histological images. Instead of treating spatial domain identification as a purely clustering task, ProST aims to recover spatial regions that are consistent with underlying biological structures, thereby aligning optimization with biological interpretability. To this end, ProST reformulates histological images as morphology-aware prompts that provide structured and denoised contextual guidance, rather than directly fusing them with gene expression. ProST was evaluated on diverse tissues, including human dorsolateral prefrontal cortex, human breast cancer, mouse brain sections, HER2+ breast tumors, and chicken heart sections at multiple developmental stages. Across these varied settings, ranging from regular laminar structures to highly heterogeneous tumors and complex anatomy, ProST consistently achieved superior spatial domain identification.

Despite these advantages, ProST still has limitations: the computational overhead of the Transformer-based attention module when processing large numbers of spots has so far prevented its application to high-resolution ST platforms or very large spatial omics datasets. Future work will therefore focus on improving scalability and efficiency, for example through deformable attention mechanisms that attend only to relevant positions, or hierarchical graph pooling strategies that progressively reduce the number of spots while preserving spatial context. These adaptations would enable ProST to be applied to larger-scale spatial omics data.

Overall, ProST demonstrates that histological images are most effectively used not as symmetric feature sources, but as structured morphological priors that guide gene-centered representation learning, bridging the gap between predictive performance and biological interpretability.

## Supplementary Material

btag699_Supplementary_Data

## Data Availability

All datasets used in this study are publicly available at https://zenodo.org/records/22685502. Comprehensive descriptions are provided in Supplementary Text 1.

## Code availability

Source code is available at Github https://github.com/Snake-Bio/ProST.
