# Supplementary material for: ProST: an image prompt-guided multimodal representation learning framework for spatial domain identification
Source: Bioinformatics. 2026 Sep 22;42(9):btag699. doi: 10.1093/bioinformatics/btag699 (PMC13619311; doi:10.1093/bioinformatics/btag699)
Supplement: Supplementary file 1 [file btag699_supplementary_data.pdf]

Supplementary Information

ProST: an image prompt-guided multimodal representation learning framework for spatial domain identification

Chenlan Sun, Zhengxia Wang, Qingchen Zhang, Xiaodong Bai, Chong Yin, Zhipei Sang, Hongsheng Xie, Yuxing Li*

Contents

Supplementary Text **3**

Text 1. Dataset Description 3
Text 2. Evaluation Metrics 7
Text 3. Patch Extraction and Feature Construction 10
Text 4. Hyperparameter Settings 14
Text 5. Robustness to Staining Variations 18

Supplementary Figures **20**

Figure S1 20
Figure S2 21
Figure S3 22
Figure S4 23
Figure S5 24
Figure S6 25
Figure S7 26
Figure S8 27
Figure S9 28
Figure S10 29
Figure S11 30
Figure S12 31
Figure S13 32
Figure S14 33
Figure S15 34
Figure S16 35
Figure S17 36
Figure S18 37
Figure S19 38
Figure S20 39
Figure S21 40

References **41**

Supplementary Text

Text 1. Dataset Description

This study evaluates ProST on five publicly available spatial transcriptomics (ST) datasets with matched histological images, including human dorsolateral prefrontal cortex (DLPFC)[1], human breast cancer (HBC)[2], mouse brain tissue sections (anterior sagittal, posterior sagittal, and coronal; MBA, MBP, and MBC)[3], HER2-positive breast tumors (HER2+)[4], and chicken heart sections (developmental days 7, 10, and 14)[5]. These datasets cover both normal and diseased tissues, relatively regular laminar structures, highly heterogeneous tumor microenvironments, and non-mammalian cardiac tissue. In particular, the mouse brain dataset represents a tissue with high regional complexity and fine anatomical structures, while the chicken heart dataset extends the evaluation to non-mammalian cardiac tissue. Together, these datasets provide a comprehensive benchmark for evaluating spatial domain identification methods.

1. DLPFC Dataset

The human dorsolateral prefrontal cortex (DLPFC) dataset was generated from post-mortem human brain tissue sections and is widely used as a benchmark dataset for spatial domain identification. The dataset contains 12 tissue slices collected from three individuals, with each individual contributing four spatial transcriptomics slices. Each slice contains thousands of spatial spots, with the number of spots ranging from approximately 3,460 to 4,789.

The DLPFC dataset is particularly suitable for evaluating the ability of computational methods to identify fine-grained spatial domains because the human cortex has a well-organized laminar structure. The original study manually annotated the tissue sections into spatial domains corresponding to cortical layers and white matter. Depending on the slice, the manual annotations include 5–7 regions. These annotations provide reliable reference labels for quantitative evaluation of clustering performance.

In this study, the DLPFC dataset is used to evaluate whether ProST can accurately recover cortical layers with subtle boundaries and different thicknesses. In particular, thin cortical layers such as layers 1, 2, and 4 provide challenging cases for spatial domain identification. A method with strong spatial resolution should preserve the relative thickness and continuity of these layers rather than merging them with adjacent regions or artificially expanding them into thicker domains.

Data access: The DLPFC dataset is publicly available through the spatialLIBD resource:

`http://spatial.libd.org/spatialLIBD`

2. HBC Dataset

The human breast cancer (HBC) dataset is used to evaluate the performance of ProST in a complex tumor microenvironment. Compared with the DLPFC dataset, which contains relatively regular cortical layers, the HBC dataset exhibits highly heterogeneous histological structures, including tumor regions, non-tumor regions, stromal components, and transitional areas at tumor boundaries.

In this study, the HBC dataset corresponds to a spatial transcriptomics section of human breast cancer tissue with matched H&E staining. The dataset contains 3,798 spatial spots. The tissue was manually annotated into 20 spatial domains based on histopathological features and spatial gene expression patterns. These annotations include major breast cancer-associated morphotypes, such as ductal carcinoma in situ/lobular carcinoma in situ (DCIS/LCIS), healthy tissue, invasive ductal carcinoma (IDC), and tumor-edge regions with relatively low malignant features. These major regions were further subdivided into finer subregions, reflecting the spatial heterogeneity of the breast cancer microenvironment.

This dataset is particularly useful for assessing whether a method can identify multi-scale tumor organization. A robust spatial domain identification method should not only separate broad pathological compartments, such as tumor and non-tumor regions, but also reveal finer intratumoral substructures within histologically similar regions. Therefore, the HBC dataset provides an important benchmark for evaluating the ability of ProST to resolve tumor heterogeneity and identify biologically meaningful subdomains.

Data access: The HBC dataset is publicly available from the 10× Genomics spatial gene expression dataset portal:

`https://support.10xgenomics.com/spatial-gene-expression/datasets`

3. Mouse Brain Dataset

The mouse brain dataset used in this study is derived from the 10x Genomics Visium platform and consists of three tissue sections: a sagittal anterior section (MBA), a sagittal posterior section (MBP), and a coronal section (MBC). These sections contain 2,695, 3,355, and 6,050 spatial spots, respectively, and provide complementary anatomical views of the mouse brain.

The MBA dataset consists of a single anterior mouse brain slice with matched histological image information and contains 2,695 spatial spots. It has detailed manual annotations covering 52 known anatomical clusters and one unmarked region. These annotations include multiple brain regions with distinct biological functions and spatial organization. Compared with laminar cortical tissue, the mouse brain contains more complex and irregular anatomical structures, including olfactory bulb regions, cortical regions, and other fine-grained substructures. Therefore, the MBA dataset provides a

challenging benchmark for evaluating whether spatial domain identification methods can recover complex anatomical boundaries. In this study, the MBA dataset is used to examine whether ProST can distinguish both large-scale anatomical regions and fine-grained brain subregions. Particular attention is given to structurally complex regions, such as the main olfactory bulb and cortical layer-related regions.

In contrast, the MBP and MBC sections do not have manual annotations. For these two sections, we use the Silhouette Coefficient (SC) and Davies–Bouldin (DB) index to evaluate the quality of the identified spatial domains without reference labels. These unsupervised metrics assess the compactness and separation of the clustering results, providing complementary evidence for the biological coherence of the spatial domains.

Accurate identification of these regions across all three sections requires effective integration of gene expression, spatial location, and histological morphology, consistent with the design goal of ProST.

Data access: The mouse brain dataset is publicly available from the 10x Genomics dataset resource:

`https://www.10xgenomics.com/resources/datasets`

4. HER2+ Dataset

The HER2-positive breast tumor dataset, referred to as the HER2+ dataset in this study, is used to evaluate the generalization ability of ProST on breast tumor tissues generated from a Visium-like spatial transcriptomics platform. The dataset contains eight tumor tissue slices, each derived from a different individual with HER2-positive breast cancer.

Compared with the HBC dataset, the HER2+ dataset contains relatively small spatial transcriptomics sections. The number of spots per slice ranges from 176 to 613. Despite the smaller number of spots, the dataset contains clear histological heterogeneity and manually annotated spatial domains. Each slice was annotated into 4–7 tissue regions, including tumor-associated and non-tumor-associated areas. These annotations provide reference labels for evaluating spatial domain identification performance.

The HER2+ dataset is useful for assessing whether ProST can accurately delineate tumor regions, connective tissue, adipose tissue, and other spatially organized tissue compartments across multiple patient-derived tumor sections. Because the histological images contain visible morphological differences among tissue regions, this dataset also provides an opportunity to evaluate whether morphology-aware image prompts can improve spatial domain identification.

Data access: The HER2+ dataset is publicly available at:

`https://github.com/almaan/her2st?tab=readme-ov-file`

5. Chicken Heart Dataset

The chicken heart dataset is used to evaluate the ability of ProST to identify spatial domains in cardiac tissue and to assess its generalization across non-mammalian species. The dataset consists of three chicken heart sections collected at developmental days 7, 10, and 14, containing 535, 969, and 1,967 spatial spots, respectively. These three developmental stages reflect progressive morphological and functional maturation of the heart.

Each section has manual annotations covering major cardiac regions, including the trabecular left ventricle (LV), endocardium, atria, and right ventricle. These annotated regions provide reference labels for quantitative evaluation of spatial domain identification. Compared with mammalian brain and tumor tissues, avian cardiac tissue exhibits distinct histological organization and developmental dynamics, offering a complementary benchmark for testing cross-species and cross-organ generalization.

In this study, the chicken heart dataset is used to examine whether ProST can accurately delineate anatomically distinct cardiac compartments and their boundaries across different developmental stages. Because the tissue sections contain clear morphological differences among regions, this dataset also provides an opportunity to evaluate whether morphology-aware image prompts can improve spatial domain identification in a non-mammalian context.

Data access: The chicken heart dataset is publicly available at:
`https://zenodo.org/records/4517120`

Overall, these five datasets provide complementary evaluation scenarios. The DLPFC dataset evaluates the recovery of fine cortical layers in human brain tissue; the HBC dataset evaluates multiscale tumor microenvironment organization; the mouse brain dataset (MBA, MBP, and MBC) evaluates regionally complex and anatomically fine-grained brain structures across anterior, posterior, and coronal sections; the HER2+ dataset evaluates spatial domain identification across multiple HER2-positive breast tumor sections; and the chicken heart dataset evaluates cross-species and cross-organ generalization in developing cardiac tissue. Together, they provide a comprehensive benchmark for assessing the robustness, accuracy, and generalizability of ProST.

Text 2. Evaluation Metrics

To quantitatively evaluate the performance of spatial domain identification, we used four complementary clustering evaluation metrics. For datasets with reference annotations, we used the Adjusted Rand Index (ARI) and the Normalized Mutual Information (NMI), which compare the predicted spatial domain labels with the reference annotations. For datasets without reference annotations, such as the MBP and MBC mouse brain sections, we used the Silhouette Coefficient (SC) and the Davies-Bouldin (DB) index, which assess clustering quality based on the compactness and separation of the identified spatial domains.

Adjusted Rand Index

The Adjusted Rand Index (ARI) measures the similarity between two clustering results by considering all pairs of samples and determining whether each pair is assigned consistently in the predicted and reference partitions. Compared with the original Rand Index, ARI corrects for the similarity that may occur by random chance, making it more suitable for evaluating clustering performance.

Given a set of samples, let $U = \{U_1, U_2, \dots, U_R\}$ denote the reference partition and $V = \{V_1, V_2, \dots, V_C\}$ denote the predicted partition. Let n_{ij} be the number of samples shared by reference cluster U_i and predicted cluster V_j. The contingency table is denoted as $\{n_{ij}\}$, where

$$a_i = \sum_{j=1}^{C} n_{ij}, \quad b_j = \sum_{i=1}^{R} n_{ij}. \tag{1}$$

Here, a_i and b_j are the marginal sums of the contingency table, and n is the total number of samples. The ARI is defined as

$$\text{ARI} = \frac{\sum_{ij} \binom{n_{ij}}{2} - \frac{\sum_i \binom{a_i}{2} \sum_j \binom{b_j}{2}}{\binom{n}{2}}}{\frac{1}{2}\left[\sum_i \binom{a_i}{2} + \sum_j \binom{b_j}{2}\right] - \frac{\sum_i \binom{a_i}{2} \sum_j \binom{b_j}{2}}{\binom{n}{2}}}. \tag{2}$$

The value of ARI generally ranges from -1 to 1. A value close to 1 indicates strong agreement between the predicted clustering and the reference annotation, while a value close to 0 indicates performance comparable to random assignment. Negative values suggest agreement worse than random expectation.

Normalized Mutual Information

Normalized Mutual Information (NMI) evaluates the agreement between two partitions from an information-theoretic perspective. It measures how much information is shared

between the predicted labels and the reference labels. A higher NMI value indicates that the predicted spatial domains preserve more information from the reference annotations.

Let U and V denote the reference and predicted partitions, respectively. The mutual information between U and V is defined as

$$I(U,V) = \sum_{i=1}^{R}\sum_{j=1}^{C} \frac{n_{ij}}{n} \log\left(\frac{n \cdot n_{ij}}{a_i b_j}\right). \tag{3}$$

Here, n_{ij} is the number of samples shared by cluster U_i and cluster V_j, a_i and b_j are the marginal sums of the contingency table, and n is the total number of samples.

The entropy of the reference partition U is defined as

$$H(U) = -\sum_{i=1}^{R} \frac{a_i}{n} \log\left(\frac{a_i}{n}\right). \tag{4}$$

The entropy of the predicted partition V is defined as

$$H(V) = -\sum_{j=1}^{C} \frac{b_j}{n} \log\left(\frac{b_j}{n}\right). \tag{5}$$

NMI is then calculated as

$$\mathrm{NMI}(U,V) = \frac{2I(U,V)}{H(U) + H(V)}. \tag{6}$$

The value of NMI ranges from 0 to 1. A value of 1 indicates perfect agreement between the predicted clustering and the reference annotation, whereas a value close to 0 indicates little shared information between the two partitions. In this study, higher ARI and NMI values indicate better spatial domain identification performance for datasets with reference annotations.

Silhouette Coefficient

The Silhouette Coefficient (SC) is an unsupervised metric that evaluates the quality of a clustering result by measuring how similar each sample is to its own cluster compared with other clusters. It does not require reference labels, making it suitable for datasets such as the MBP and MBC mouse brain sections, which lack manual annotations.

For each sample i, let $a(i)$ be the average distance between i and all other samples in the same cluster, and let $b(i)$ be the minimum average distance between i and all samples in any other cluster. The silhouette score for sample i is defined as

$$s(i) = \frac{b(i) - a(i)}{\max\{a(i), b(i)\}}. \tag{7}$$

The Silhouette Coefficient for the entire dataset is the average of $s(i)$ over all samples:

$$\text{SC} = \frac{1}{n}\sum_{i=1}^{n} s(i). \tag{8}$$

The value of SC ranges from −1 to 1. A value close to 1 indicates that the clusters are compact and well separated, a value close to 0 indicates overlapping clusters, and negative values suggest that samples may have been assigned to the wrong clusters. In this study, higher SC values indicate better spatial domain identification performance for unannotated datasets.

Davies-Bouldin Index

The Davies-Bouldin (DB) index is another unsupervised metric that evaluates clustering quality by measuring the average similarity between each cluster and its most similar cluster. Lower DB values indicate better clustering, as they reflect more compact and well-separated clusters.

Let k be the number of clusters. For each cluster i, let S_i denote the average distance between the samples in cluster i and the centroid of cluster i, which measures the within-cluster dispersion. Let M_{ij} denote the distance between the centroids of clusters i and j. The similarity between clusters i and j is defined as

$$R_{ij} = \frac{S_i + S_j}{M_{ij}}. \tag{9}$$

For each cluster i, we compute its maximum similarity to any other cluster:

$$R_i = \max_{j \neq i} R_{ij}. \tag{10}$$

The Davies-Bouldin index is then defined as the average of these maximum similarities over all clusters:

$$\text{DB} = \frac{1}{k}\sum_{i=1}^{k} R_i. \tag{11}$$

The value of DB is non-negative, and lower values indicate better clustering performance. In this study, we use the DB index to evaluate the compactness and separation of spatial domains in unannotated datasets such as MBP and MBC, where lower DB values correspond to more biologically coherent spatial domains.

Text 3. Patch Extraction and Feature Construction

Given a spatial transcriptomics (ST) dataset with N spatial spots, ProST uses three complementary types of input information: gene expression profiles, spatial coordinates, and histological image patches. The gene expression profiles provide molecular information, the spatial coordinates preserve the physical organization of spots on the tissue section, and the histological image patches provide local morphological context around each spatial spot.

For histological image preprocessing, an image patch was cropped for each spatial spot from the matched H&E-stained whole-slide image. Each patch was centered at the spatial coordinate of the corresponding spot. Because the size of the cropped image patch determines how much morphological context is introduced into the model, the patch cropping strategy is an important factor affecting the quality of image-derived features.

We designed and evaluated four single-scale patch cropping strategies according to two dataset-specific geometric quantities: the spot diameter D and the nearest-neighbor spot center distance d. Here, D represents the diameter of a spatial spot projected onto the histological image, while d represents the median distance between the centers of neighboring spots. These two quantities jointly describe the physical scale of each spot and the spacing between adjacent spots in the image coordinate system. The comparison of these strategies is shown in Supplementary Fig. S1.

Strategy 1: Spot-diameter-based patch

The first strategy uses the spot diameter as the patch side length:

$$S_1 = D. \tag{12}$$

This strategy crops an image patch that mainly covers the morphology within the spot itself, providing a direct morphological representation associated with the measured gene expression profile. In the default setting of ProST, this strategy is used for histological image feature extraction to focus on spot-specific morphology while minimizing interference from neighboring spots.

Strategy 2: Non-overlapping local patch

The second strategy uses a slightly larger, non-overlapping local patch size $S_2 = d$, calculated based on the dataset's spot arrangement. Compared with Strategy 1, it captures additional local morphology surrounding each spot while still reducing the risk of mixing signals from neighboring spots.

Strategy 3: Neighbor-center-avoiding contextual patch

The third strategy uses a patch size that maximizes contextual tissue information while avoiding inclusion of neighboring spot centers:

$$S_3 = 2d - D. \tag{13}$$

This strategy captures broader morphological neighborhoods and is useful when the surrounding tissue contributes meaningful information, while preserving a relatively clear correspondence between the patch and the target spot.

Strategy 4: Broad-context patch

The fourth strategy uses a broader tissue context with side length:

$$S_4 = 2d. \tag{14}$$

This captures a wider tissue region around each spot, which is important for recognizing extended histological structures such as cortical layers, tumor boundaries, or stromal regions. However, it may introduce information from neighboring spatial domains.

Strategy 5: Multi-scale concatenated image features

Inspired by the success of multi-scale features in other domains, we introduced a fifth strategy that combines the four single-scale strategies. In this approach, image patches from scales S_1–S_4 are independently resized to 224×224 pixels and encoded using the same histopathology image encoder. The resulting feature vectors are then concatenated and projected via PCA to a 1,024-dimensional representation:

$$\mathbf{f}_i^{multi} = \mathrm{PCA}_{1024}\left([\mathbf{f}_i^{(1)}; \mathbf{f}_i^{(2)}; \mathbf{f}_i^{(3)}; \mathbf{f}_i^{(4)}]\right), \tag{15}$$

where $\mathbf{f}_i^{(m)}$ denotes the feature of spot i from the m-th scale. This multi-scale strategy allows the model to leverage spot-specific, local, neighbor-aware, and broad morphological information simultaneously, while controlling feature dimensionality and avoiding redundancy.

Comparison of patch strategies

Among the five patch extraction strategies, Strategy 1 (spot-diameter-based patch) achieved the best spatial domain identification performance. The evaluation was conducted on 14 tissue sections, including 12 DLPFC slices, the HBC section, and the MBA section, thereby covering diverse tissue types, anatomical structures, and staining conditions. The multi-scale strategy (Strategy 5) was included as a supplementary comparison to

investigate whether combining multiple patch sizes could further improve spatial domain identification. The results indicate that the spot-matched patch (Strategy 1) provides the most effective morphological information for integration with gene expression and spatial information. This finding is consistent with the biological interpretation that each spot's local morphology should correspond directly to its measured gene expression profile, whereas larger patches may introduce signals from neighboring spots and dilute spot-specific morphological features. The performance comparison is shown in Supplementary Fig. S1.

Dataset-specific patch sizes

For each dataset, the crop sizes for scales S_1–S_4 were set based on spot diameter and nearest-neighbor distance, including the DLPFC, HBC, mouse brain (MBA, MBP, MBC), HER2+, and chicken heart datasets. After cropping, all patches were resized to 224×224 pixels and encoded using the Universal Histopathology Image Encoder (UNI)[6]. The UNI encoder was selected as the default feature extractor after comparing several histopathology image encoders, including UNI[6], UNI2-h[6], CONCH[7], ResNet-50[8], and Inception-v3[9]. This comparison was also performed on the same 14 tissue sections (DLPFC, HBC, and MBA), ensuring that the selected encoder is robust across different biological tissues, disease regions, and staining environments. As shown in Supplementary Fig. S2, UNI achieved the best performance when combined with ProST, and therefore was used as the default patch feature extractor in all subsequent experiments.

UNI has been trained on a large-scale and diverse collection of histopathology images spanning multiple tissue types and various staining conditions, which provides strong generalization ability for patch-level feature extraction. Therefore, in our spot-centered patch setting, UNI can already produce sufficiently discriminative local morphological representations. Although UNI2-h is a newer and larger model, its extracted features performed relatively worse in this task. Several factors may account for this. First, domain mismatch: UNI2-h was trained on a broader range of organs and staining protocols, but our task uses fixed tissue types, H&E staining, and small spot-centered patches; in this narrower setting, the broader training distribution may not improve fine-grained local features. Second, over-parameterization: the additional capacity of UNI2-h may not be fully utilized on relatively homogeneous patch inputs, potentially leading to redundant or less stable features. Third, differences in pre-training objectives and data curation may favor generalization across large-scale heterogeneous datasets rather than fine-grained local morphology in a single modality, which could reduce feature consistency in our specific context. These observations may be helpful for researchers selecting feature extractors for similar spot-level histopathology tasks.

The resulting feature is a 1,024-dimensional vector per spot for single-scale strategies,

while the multi-scale strategy uses the PCA-reduced 1,024-dimensional representation.

To incorporate spatial location, a k-nearest-neighbor (KNN) graph was constructed from spot coordinates, producing an undirected graph $\mathcal{G} = (\mathcal{V}, \mathcal{E})$ with adjacency matrix $\mathbf{A} \in \mathbb{R}^{N \times N}$. In our implementation, we set $k = 6$ by default. This spatial graph provides neighborhood context for downstream modules. By combining gene expression, image patch features, and spatial graph information, ProST constructs a multimodal representation integrating molecular, morphological, and spatial information for accurate spatial domain identification.

Text 4. Hyperparameter Settings

To determine suitable model configurations for ProST, we tested four key hyperparameters, including the prompt pool size, the number of retrieved prompts, the gate ratio, and the masking ratio. The corresponding sensitivity analyses were conducted on three annotated datasets with reference labels for quantitative evaluation: the DLPFC dataset (12 slices), the HBC dataset, and the MBA dataset. For each hyperparameter setting, we evaluated spatial domain identification performance using ARI and NMI, and inspected the resulting spatial domain visualizations. The quantitative results are shown in Supplementary Figs. S14 and S15. To further examine how the prompt pool size (M) and the number of retrieved prompts (top-K) jointly influence the final spatial domain assignments, we also visualized the spatial domain identification results on representative sections under different combinations of these two hyperparameters (Supplementary Figs. S16–S18). These visualization results further demonstrate that the default settings ($M = 16$, $K = 3$) consistently yield spatial domains that best match the manual annotations across all three tissue types, including regular cortical layers and complex tumor microenvironments, indicating that a moderate number of morphological prototypes with sparse retrieval is sufficient for accurate spatial domain identification across diverse tissue architectures.

Prompt pool size

The first hyperparameter is the prompt pool size, denoted as M, which controls the number of learnable key-value prompt components maintained in the Image Prompt Pool (IPP). A smaller prompt pool may have insufficient capacity to represent diverse histological morphology patterns, whereas an excessively large prompt pool may introduce redundant prompt components and increase optimization difficulty. In ProST, the prompt pool size was selected by comparing spatial domain identification performance under different values of M.

To investigate whether different tissue types require different numbers of morphological prototypes, we scanned M across a wide range (5–35, 50, 64, 100, 150, 200, 250, 256 and 300) on the DLPFC, HBC, and MBA datasets separately. As shown in Supplementary Fig. S14A, the model achieved its best average performance around $M = 16$ on the DLPFC dataset, which contains relatively regular cortical layers. On the HBC dataset, characterized by a highly heterogeneous tumor microenvironment with multiple fine subregions, the optimal performance was also observed around $M = 16$, although a slightly larger range ($M = 11$–25) maintained comparable results, suggesting that a moderate number of prototypes is sufficient to capture the morphological diversity of tumor subregions without overfitting. On the MBA dataset, which includes anatomically complex and irregular brain structures, the best performance was likewise obtained near

$M = 16$. Overall, ProST maintained stable performance within a broad intermediate range (e.g., $M = 11$–35) across all three datasets. In contrast, extremely small values (e.g., $M = 5$–10) or overly large values (e.g., $M \geq 100$) led to a clear performance degradation, indicating that an appropriate prompt capacity is crucial for balancing representational richness and optimization stability. These results demonstrate the cross-tissue and cross-species generalization capability of ProST, as well as its good robustness. The final setting provides a balance between morphology-aware representation capacity and model stability. In our implementation, the default prompt pool size was set to $M = 16$.

To further examine the influence of M on spatial domain assignments, we visualized the resulting spatial domains on three representative sections: DLPFC slice 151675, the HBC section, and the MBA section (Supplementary Figs. S16–S18). Across all three tissues, smaller values of M (e.g., $M = 8$) produced over-fragmented domains, especially in the HBC dataset where multiple small clusters appeared within histologically homogeneous regions. Conversely, larger values of M (e.g., $M = 256$) led to over-smoothing, merging adjacent but biologically distinct regions such as cortical layers or tumor subregions. In contrast, the default $M = 16$ consistently yielded spatial domains that best matched the reference annotations, with clear boundaries and appropriate granularity. These visual results align with the ARI and NMI trends and confirm that a shared prompt pool of moderate size can effectively capture tissue-specific morphological prototypes without requiring dataset-specific tuning.

Number of retrieved prompts

The second hyperparameter is the number of retrieved prompts, denoted as top-K. For each spot, IPP retrieves the K most relevant prompts from the shared prompt pool according to the similarity between the refined image token and the prompt keys. A small K may restrict the diversity of morphology-aware guidance, while a large K may introduce weakly related or noisy prompt components. Therefore, top-K determines the sparsity and specificity of prompt retrieval.

We similarly scanned top-K from 1 to 15 on the DLPFC, HBC, and MBA datasets. As shown in Supplementary Fig. S14B, ProST achieved optimal performance at $K = 3$ on all three datasets, with consistently high performance observed for $K = 4$ and $K = 5$. On the DLPFC dataset, where cortical layers exhibit relatively homogeneous within-layer morphology, smaller K values were sufficient and larger K introduced slight noise. On the HBC dataset, although the tumor microenvironment is more heterogeneous, sparse retrieval with $K = 3$ still performed best, indicating that each spot's morphology can be effectively represented by a small number of highly relevant prompts rather than a broad mixture. On the MBA dataset, similar trends were observed. When K increased beyond approximately 5, a gradual performance decline was observed across all datasets, likely

due to the incorporation of less relevant prompt components that introduce noise into the fusion process. These results indicate that a sparse retrieval strategy is beneficial for preserving the most informative image-prompt components while suppressing irrelevant visual perturbations. The default value was set to $K = 3$.

Spatial domain visualizations under different top-K values are also provided in Supplementary Figs. S16–S18. With $K = 1$, the retrieved prompts sometimes failed to capture the full local morphological context, leading to discontinuous domains or missing boundaries in complex regions such as the MBA olfactory bulb or HBC tumor–stroma interfaces. Conversely, when K was larger, for example $K = 6$, the resulting domains became overly smooth, and fine structures such as thin cortical layers or small tumor subregions were merged into larger domains. At the default $K = 3$, the identified domains exhibited the clearest correspondence to manual annotations across all three tissues, preserving both coarse compartments and fine-grained boundaries. This visual evidence further supports the choice of a sparse prompt retrieval strategy and demonstrates that $K = 3$ generalizes well across different tissue architectures.

Gate ratio

The third hyperparameter is the gate ratio, denoted as η, which controls the overall contribution of the image-side representation in the Gene-centered Adaptive Fusion (GAF) module. Specifically, the final embedding is obtained by adding a gated image-side residual term to the prompt-guided gene-side representation. A small gate ratio weakens the contribution of morphology-aware information, whereas a large gate ratio may overemphasize image-derived features and reduce the dominance of gene expression. Since ProST is designed as a gene-centered multimodal framework, η was selected to allow useful histological information to complement gene expression without replacing it as the primary information source. As shown in Supplementary Fig. S15A, ProST achieved the best performance at a moderate gate ratio of $\eta = 0.5$. The performance increased when η was raised from 0.0 to 0.5, indicating that image-derived morphological cues provide effective complementary guidance for spatial representation learning. However, further increasing η led to a gradual performance decline, suggesting that excessive image-side contribution may introduce modality imbalance or visual noise and weaken the gene-centered representation. Therefore, the default gate ratio was set to $\eta = 0.5$.

Masking ratio and masking strategy

The fourth hyperparameter is the masking ratio, denoted as ρ, which determines the proportion of spots masked during masked gene modeling. In ProST, masked gene modeling follows a mixed masking strategy. A subset of spots $\mathcal{M}$ is randomly selected with probability ρ (default $\rho = 0.6$). For each masked spot, with probability 0.9 we replace

its gene features with a learnable mask embedding, and with probability 0.1 we replace them with features randomly sampled from another spot in the dataset. This mixed replacement strategy is adapted from BERT-style masking[10] and prevents the model from simply relying on the presence of a mask token, thereby encouraging it to utilize spatial and morphological context for gene reconstruction.

We systematically scanned ρ from 0.1 to 0.9 (9 values in total) on the DLPFC, HBC, and MBA datasets. As shown in Supplementary Fig. S15B, ProST achieved the best performance at $\rho = 0.6$ and maintained relatively stable performance within the range of $\rho = 0.6$–0.8. A low masking ratio (e.g., $\rho \leq 0.4$) made the reconstruction task too simple and limited the model's ability to learn robust contextual representations, while an excessively high masking ratio (e.g., $\rho = 0.9$) removed too much gene information and caused reconstruction instability, especially for sparse spatial transcriptomics data. These results indicate that a moderately strong masking strategy provides sufficient self-supervised learning difficulty while preserving enough contextual information for reliable gene reconstruction.

We further examined the effect of the random replacement probability in the mixed masking strategy. Keeping the overall masking ratio fixed at $\rho = 0.6$, we varied the probability of replacing a masked spot with random features. As shown in Supplementary Fig. S19, the clustering performance remains stable when the replacement probability is in the range of 0.05–0.2, with the best average ARI achieved at 0.1. Performance drops noticeably when the replacement probability is 0 (i.e., always using the mask embedding) or exceeds 0.3, indicating that a small amount of random replacement helps prevent shortcut learning, whereas excessive random noise disrupts the reconstruction signal. These results support our default choice of 0.1 as the random replacement probability.

Overall, these four hyperparameters affect complementary aspects of ProST: the prompt pool size controls the capacity of morphology-aware prompt representation; top-K controls prompt retrieval sparsity; the gate ratio controls the contribution of image-side residual information; and the masking ratio controls the difficulty of self-supervised gene reconstruction. Across the DLPFC, HBC, and MBA datasets, ProST maintained stable performance across a broad range of settings, demonstrating that its effectiveness is not dependent on a narrow hyperparameter configuration.

Text 5. Robustness to Staining Variations

To evaluate the robustness of ProST to variations in histological image quality, we performed both qualitative and quantitative analyses. First, we discuss the design mechanisms that inherently improve robustness, and then we present perturbation experiments that directly simulate staining batch effects.

Design-level robustness

Three aspects of ProST contribute to stability under image quality shifts. (i) The Image Prompt Pool (IPP) does not use raw image tokens directly for fusion. Instead, each image token is represented as a sparse combination of a small set of learnable morphological prototypes. This prototype-based representation acts as a soft discretization that suppresses isolated pixel- or feature-level noise and reduces sensitivity to global color and contrast changes. Because the prototypes are shared across all spots and learned during training, the retrieved prompts capture stable tissue-level morphological patterns rather than staining-specific artifacts. (ii) The image features are extracted by the Universal Histopathology Image Encoder (UNI), which was pre-trained on a large-scale collection of histopathology images spanning diverse tissue types, staining protocols, and scanning platforms. This broad pre-training distribution provides strong generalization to unseen staining variations, as also confirmed by our encoder comparison in Supplementary Fig. S2, where UNI outperformed alternative feature extractors. (iii) Our evaluation already covers multiple publicly available datasets generated by different laboratories and platforms (DLPFC, HBC, mouse brain, HER2+, and chicken heart). These datasets inherently contain different staining intensities and acquisition conditions, and ProST consistently achieved the best performance across all of them. This cross-dataset consistency provides indirect but practical evidence of robustness.

Perturbation experiments

To directly assess the impact of staining batch effects, we simulated realistic color variations by applying random perturbations to the original H&E images. Specifically, we combined brightness, contrast, saturation, and hue adjustments; then independently scaled and offset each RGB channel; and finally added Gaussian noise. The perturbation strength was controlled by a coefficient c with three levels: light ($c = 0.15$), medium ($c = 0.5$), and strong ($c = 1.0$). The detailed parameter ranges are available in the code repository.

For each dataset, we generated three perturbed versions of the original H&E image (light, medium, strong) and re-extracted image features using the same UNI encoder. Gene expression profiles and spatial coordinates were kept unchanged. We then evaluated the spatial domain identification performance of ProST on DLPFC (slice 151675), HBC,

and MBA under these perturbed image conditions. The quantitative results are shown in Supplementary Fig. S20.

As expected, the original images yielded the best overall performance, while the perturbed versions resulted in only slight and largely acceptable degradation. On DLPFC, the ARI/NMI changed from 0.68/0.69 (raw) to 0.66/0.71 (light), 0.69/0.72 (medium), and 0.63/0.67 (strong). On HBC, the values were 0.70/0.68 (raw), 0.67/0.70 (light), 0.68/0.70 (medium), and 0.64/0.67 (strong). On MBA, the ARI/NMI were 0.60/0.74 (raw), 0.55/0.71 (light), 0.51/0.69 (medium), and 0.54/0.73 (strong). Overall, the performance remained stable under light and medium perturbations; only under strong perturbations did we observe a more noticeable but still moderate decline. Even in the strong perturbation setting, the performance was clearly better than random assignment and remained competitive with many baseline methods evaluated on original images.

Qualitative observations

In addition to the quantitative results, we visually inspected the spatial domain assignments under different perturbation levels. Notably, in the DLPFC slice 151675, the original H&E image contains a clearly visible horizontal staining artifact (highlighted by a yellow box in Supplementary Fig. S20), which appears as an unevenly stained band. In routine pathological assessment, such artifacts can severely interfere with image interpretation. However, the spatial domain identification results produced by ProST under all four conditions (raw, light, medium, strong) consistently excluded this artifact region and correctly recovered the underlying laminar organization of the cortex. This observation further supports the conclusion that the prototype-based prompting mechanism and the robust UNI features enable ProST to focus on biologically meaningful tissue structures rather than superficial staining irregularities.

Collectively, these results demonstrate that ProST is robust to realistic variations in staining and scanning conditions, and that the combination of prototype-based prompting and the broadly pre-trained UNI encoder effectively mitigates the impact of image quality shifts.

Supplementary Figures

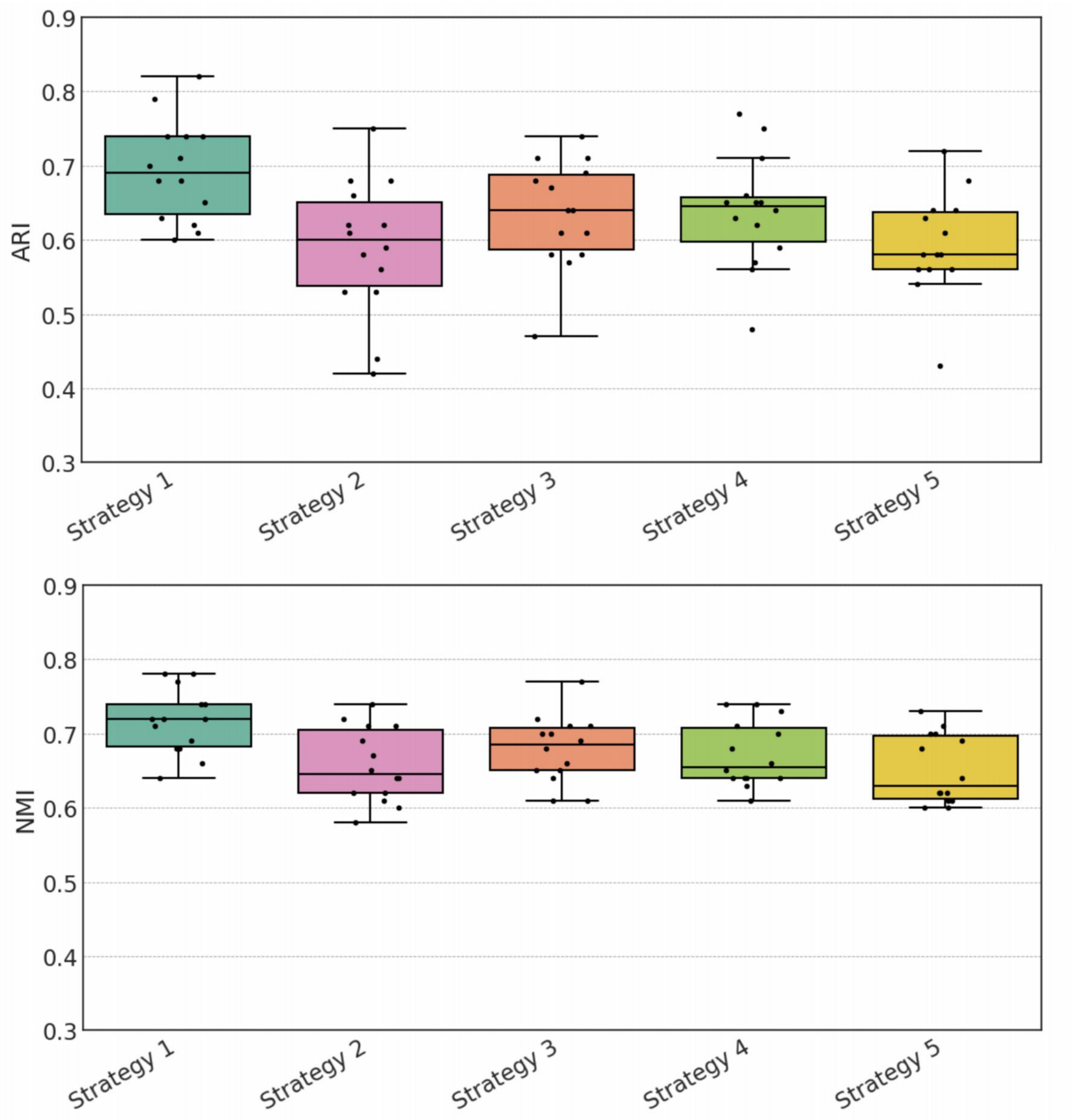

Figure S1. Comparison of different patch extraction strategies using ARI and NMI. The figure evaluates the effect of different histological patch sizes on spatial domain identification performance. Strategies 1–4 correspond to single-scale patch extraction based on spot diameter and nearest-neighbor spot distance, while Strategy 5 represents the multi-scale concatenated image feature strategy described in Text 3. Higher ARI and NMI values indicate better agreement with the reference spatial annotations.

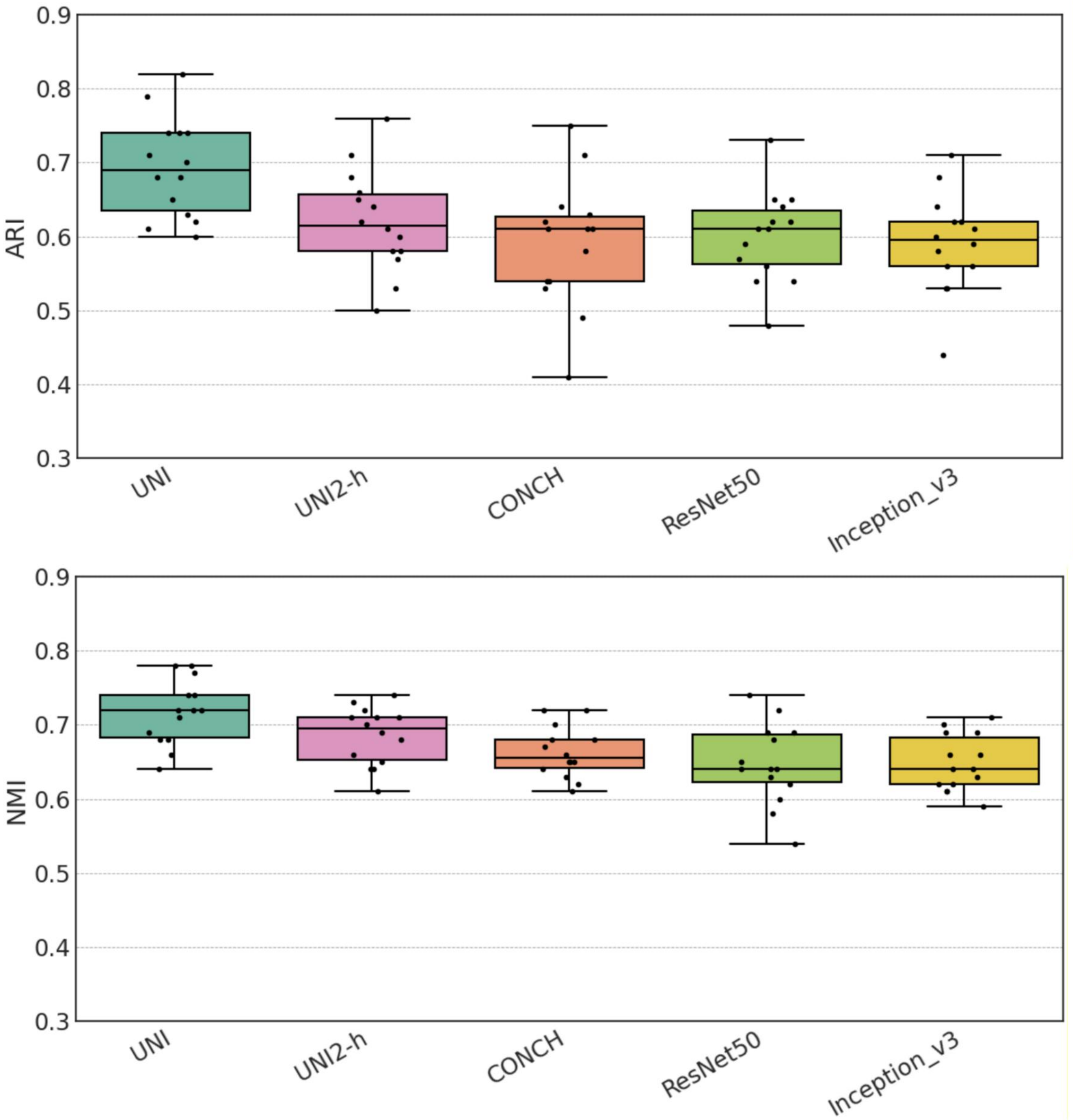

Figure S2. Comparison of different image feature extractors for histological patch representation. We evaluated UNI, UNI2-h, CONCH, ResNet50, and Inception-v3 for extracting patch-level morphological features. Among these models, UNI achieved the best performance and was therefore selected as the default patch feature extractor in ProST. A detailed discussion of the comparison and possible reasons for the inferior performance of UNI2-h is provided in Supplementary Text 3.

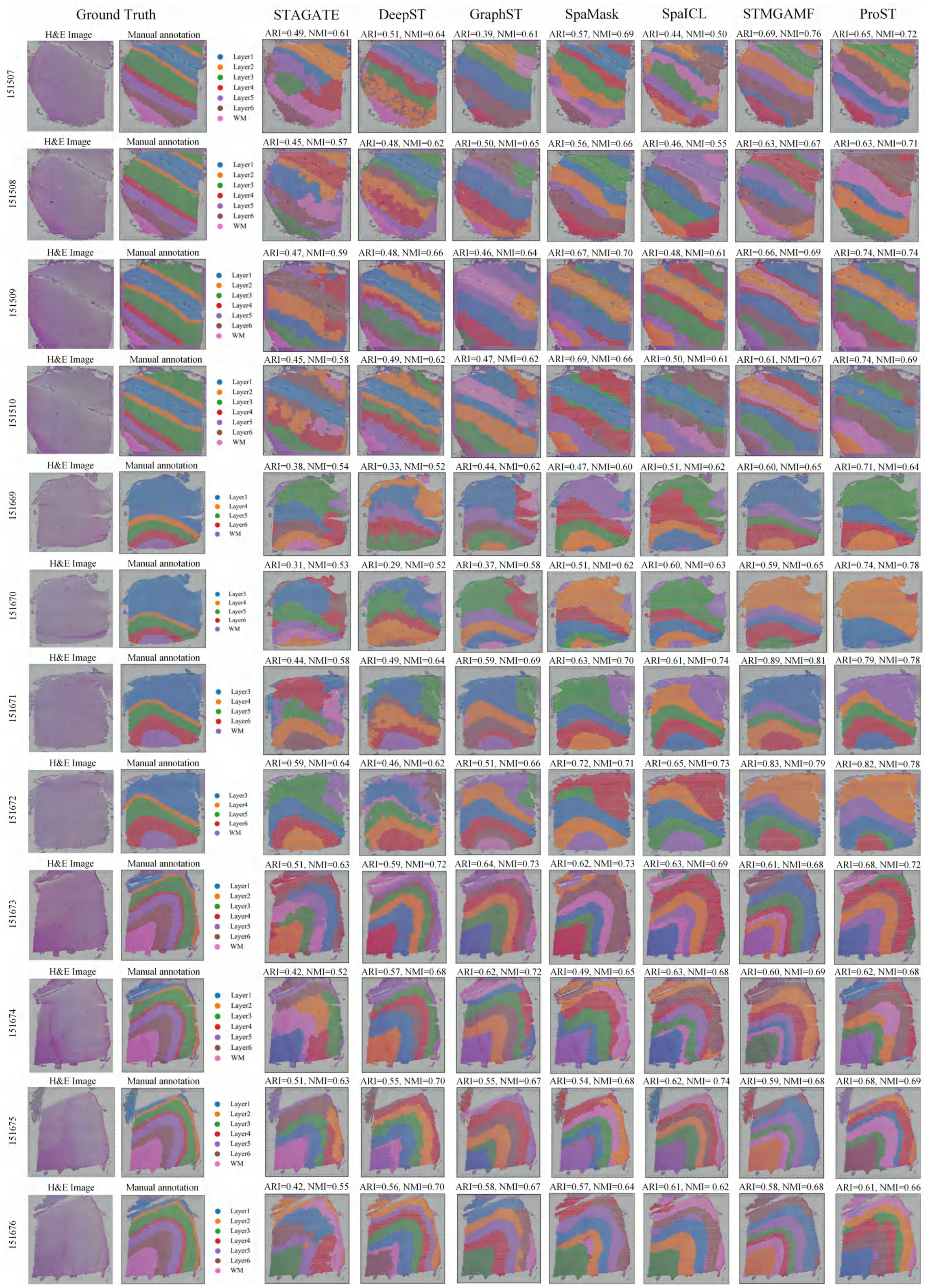

Figure S3. Comparison of spatial domain results across DLPFC slices. ProST consistently matches manual annotations and recovers cortical laminar structures. In contrast, competing methods (e.g., SpaICL and STMGAMF) with similar ARI/NMI often produce over-segmented domains with weak structural separation. Representative cases (e.g., slice 151674) show anatomical deviations, whereas ProST remains consistent across slices.

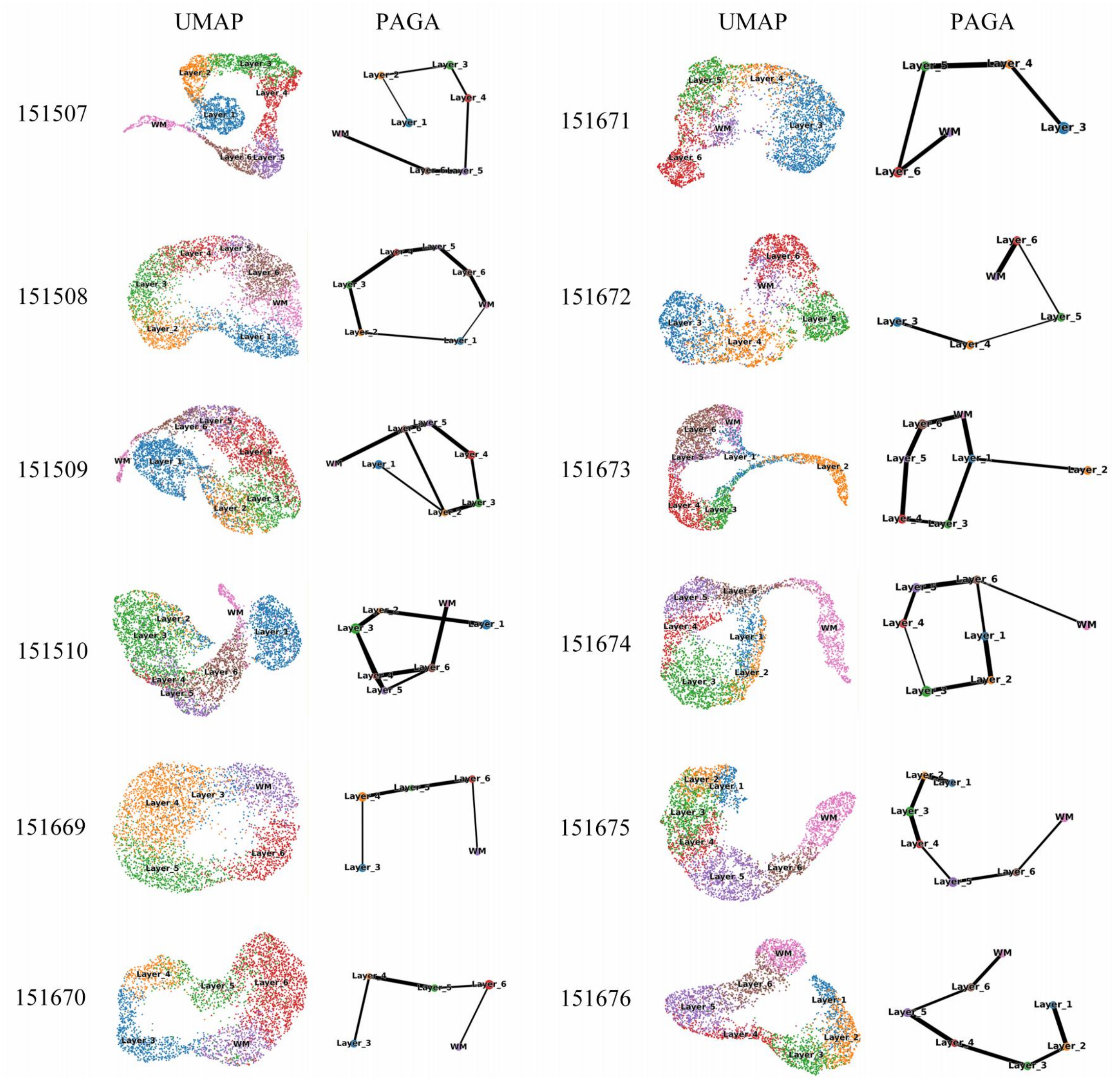

Figure S4. Results of ProST for UMAP visualization, trajectory inference across 12 slices of DLPFC datasets.

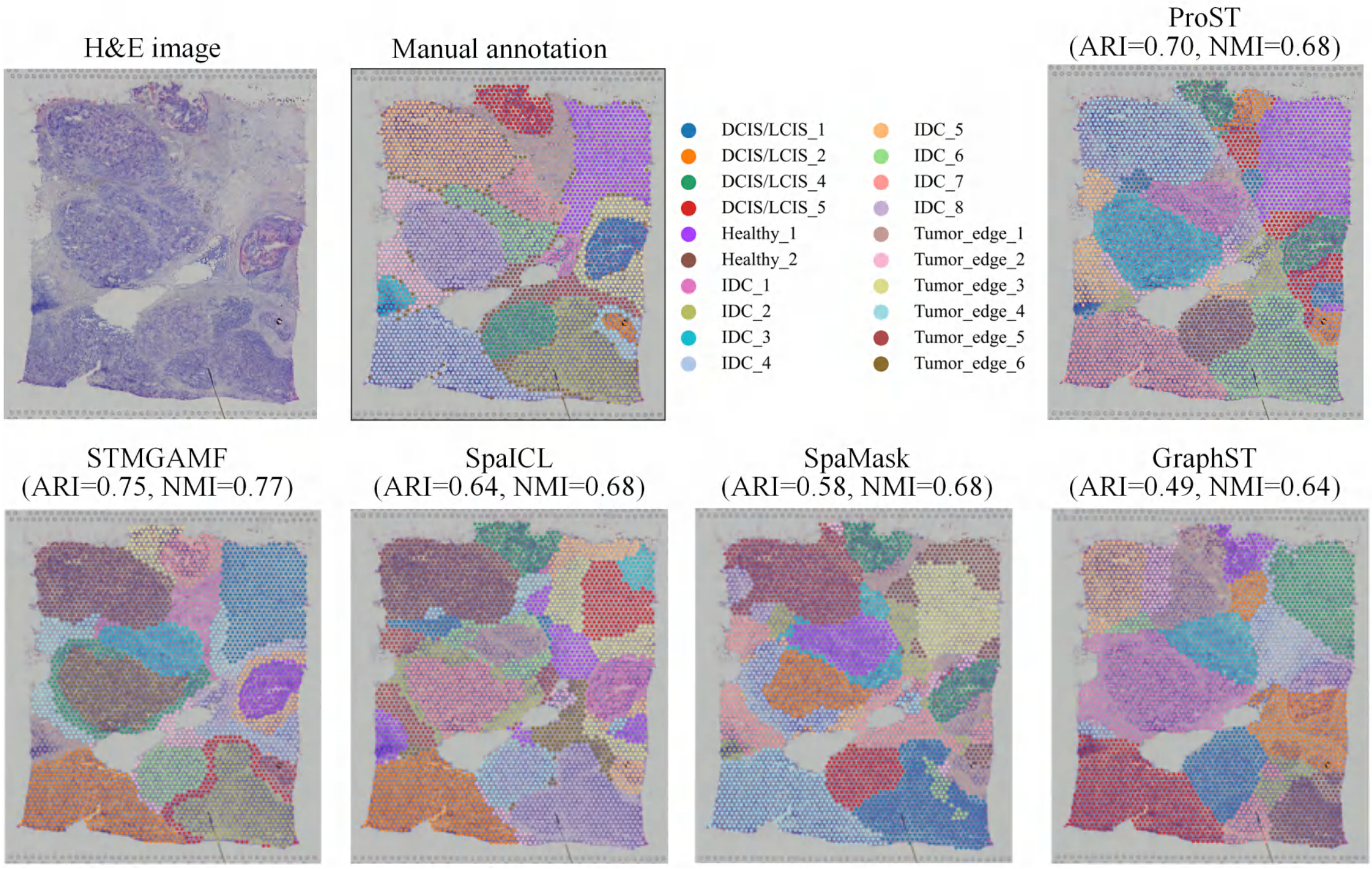

Figure S5. Comparison of spatial domain identification results on the HBC dataset. ProST outperformed most competing methods in both quantitative performance and biological consistency. Although STMGAMF achieved a slightly higher evaluation score than ProST, its spatial domains showed an over-smoothing effect, with some regions surrounded by additional domains that were inconsistent with the reference annotations. In contrast, the spatial domains identified by ProST were largely consistent with the annotated biological structures, indicating that ProST provides more biologically meaningful and spatially coherent results on the HBC dataset.

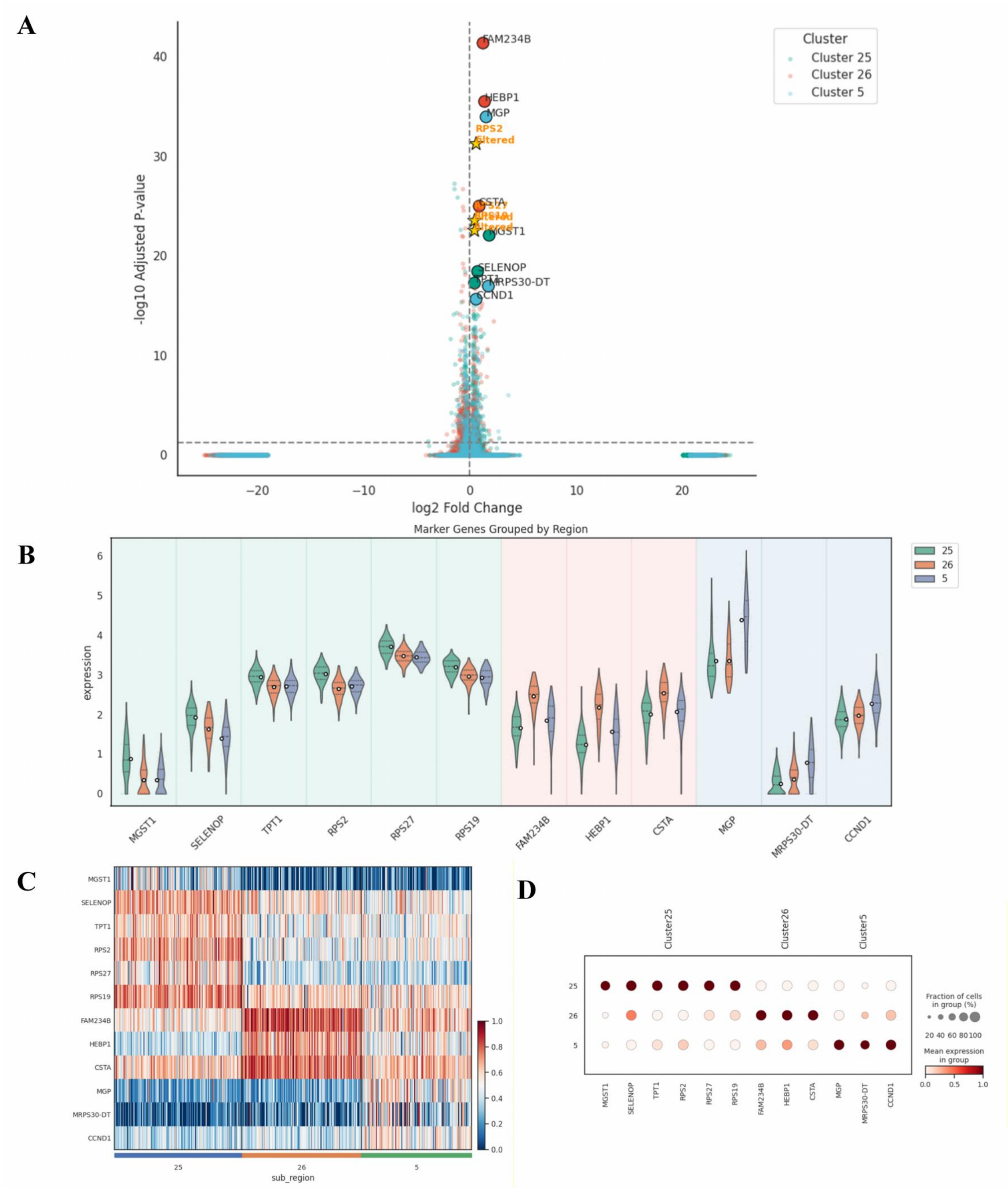

Figure S6. Differential expression and marker characterization of ProST-identified IDC_5-associated subdomains in the HBC dataset. We performed Wilcoxon-based differential expression analysis on domains 25, 26, and 5 obtained under the 28-cluster setting. Ribosomal and mitochondrial genes (RPS/RPL/MT-) were flagged when present among top-ranked markers. (A) Volcano plots showing differentially expressed genes across the three domains, with representative markers highlighted. (B) Violin plots showing expression distributions of selected marker genes across domains 25, 26, and 5. (C) Heatmap showing scaled expression patterns of selected markers across the three subdomains. (D) Dot plot summarizing marker expression, where color indicates scaled average expression and size indicates expression proportion.

Together, these results confirm the transcriptional heterogeneity of ProST-defined IDC_5 subdomains.

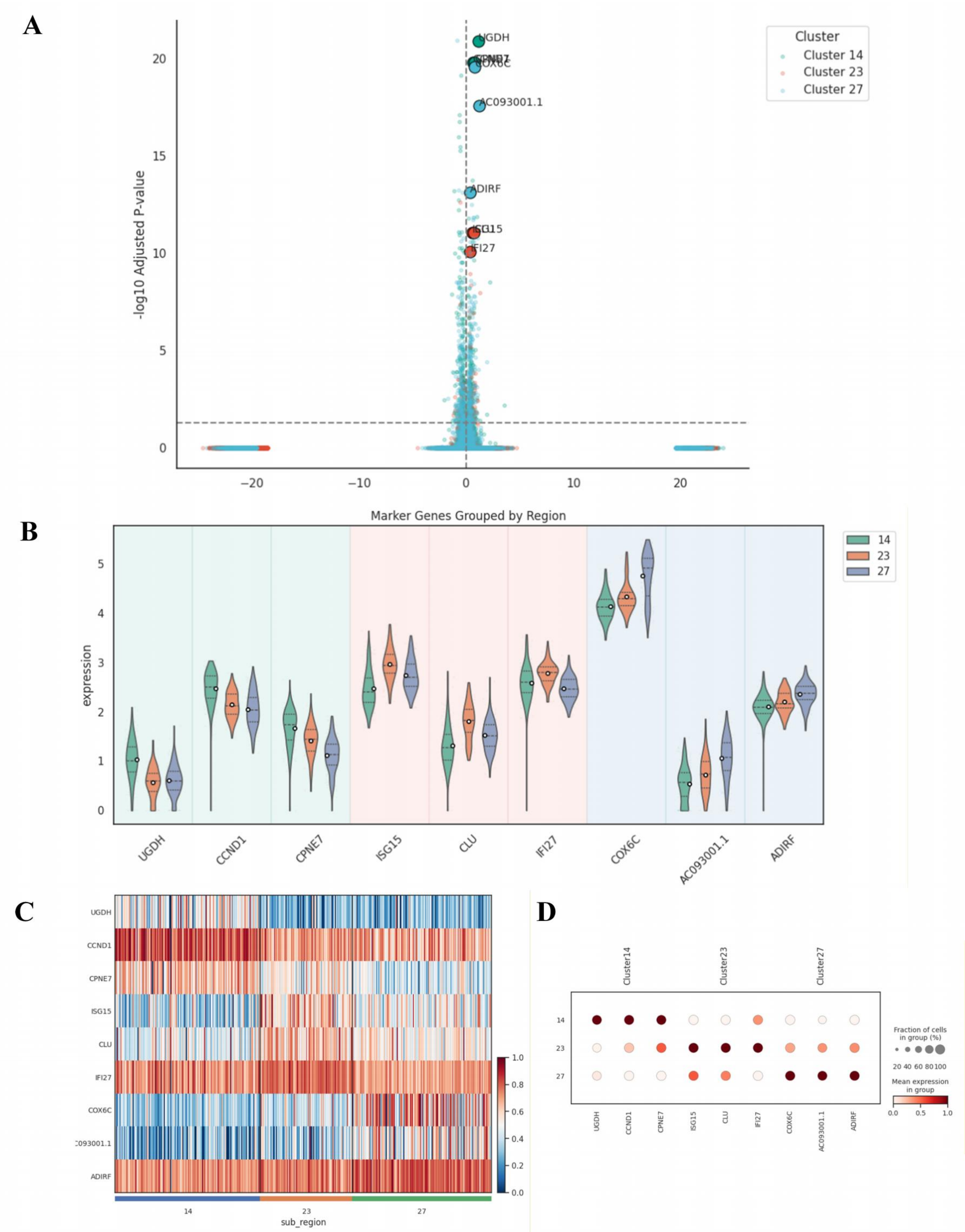

Figure S7. Differential expression and marker characterization of ProST-identified IDC_2-associated subdomains in the HBC dataset. We analyzed domains 14, 23, and 27 identified under the 28-cluster setting. (A) Volcano plots showing differentially expressed genes across the three domains. (B) Violin plots showing expression distributions of representative marker genes. (C) Heatmap showing scaled expression patterns of selected markers. (D) Dot plot summarizing marker expression patterns across the three subdomains.

These results confirm the transcriptional heterogeneity of ProST-defined IDC_2 subdomains, consistent with findings in IDC_5.

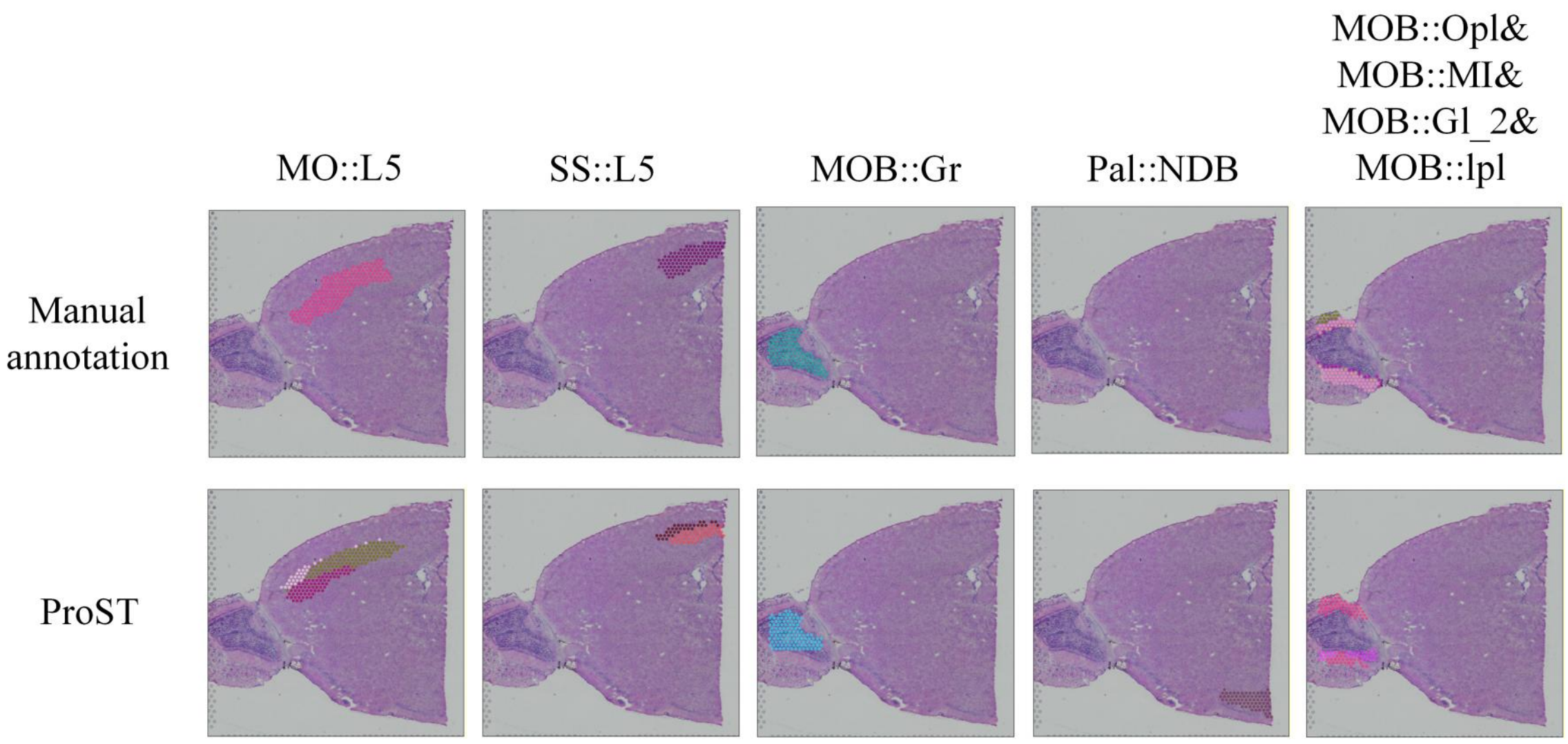

Figure S8. Spatial domain identification on the MBA dataset. ProST accurately identifies spatial domains that are highly consistent with annotated biological regions, including MO::L5, SS::L5, MOB::Gr, Pal::NDB, MOB::Opl, MOB::MI, MOB::Gl_2, and MOB::lpl. These results demonstrate that ProST can recover fine-grained anatomical structures with strong agreement to histological annotations, highlighting its robustness in capturing biologically meaningful spatial organization.

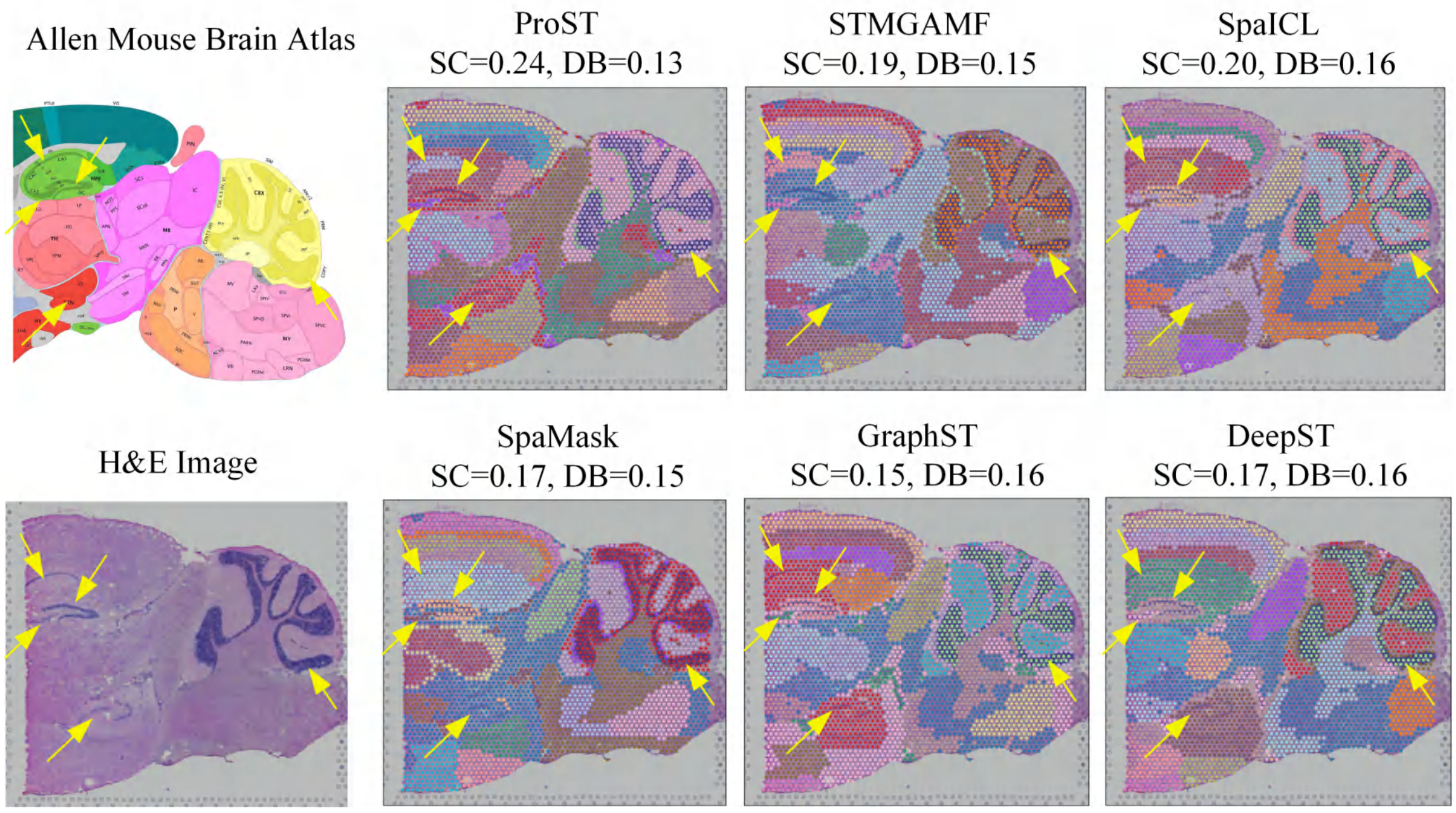

Figure S9. Spatial domain identification on the MBP dataset. In the sagittal posterior section, ProST not only resolves the clear laminar structure of the cerebellar cortex, but also accurately identifies key anatomical regions, including the hippocampus, the vermis, and the subthalamic region, yielding results highly consistent with the Allen Mouse Brain Atlas. In particular, in the hippocampus region, ProST precisely identifies four fine laminar structures, demonstrating its high-resolution identification capability, whereas none of the other compared methods can resolve this region at such fine granularity. Furthermore, although the subthalamic region is clearly visible in the H&E image, only ProST successfully delineates its structural outline among all methods, benefiting from the rational multimodal fusion design of ProST.

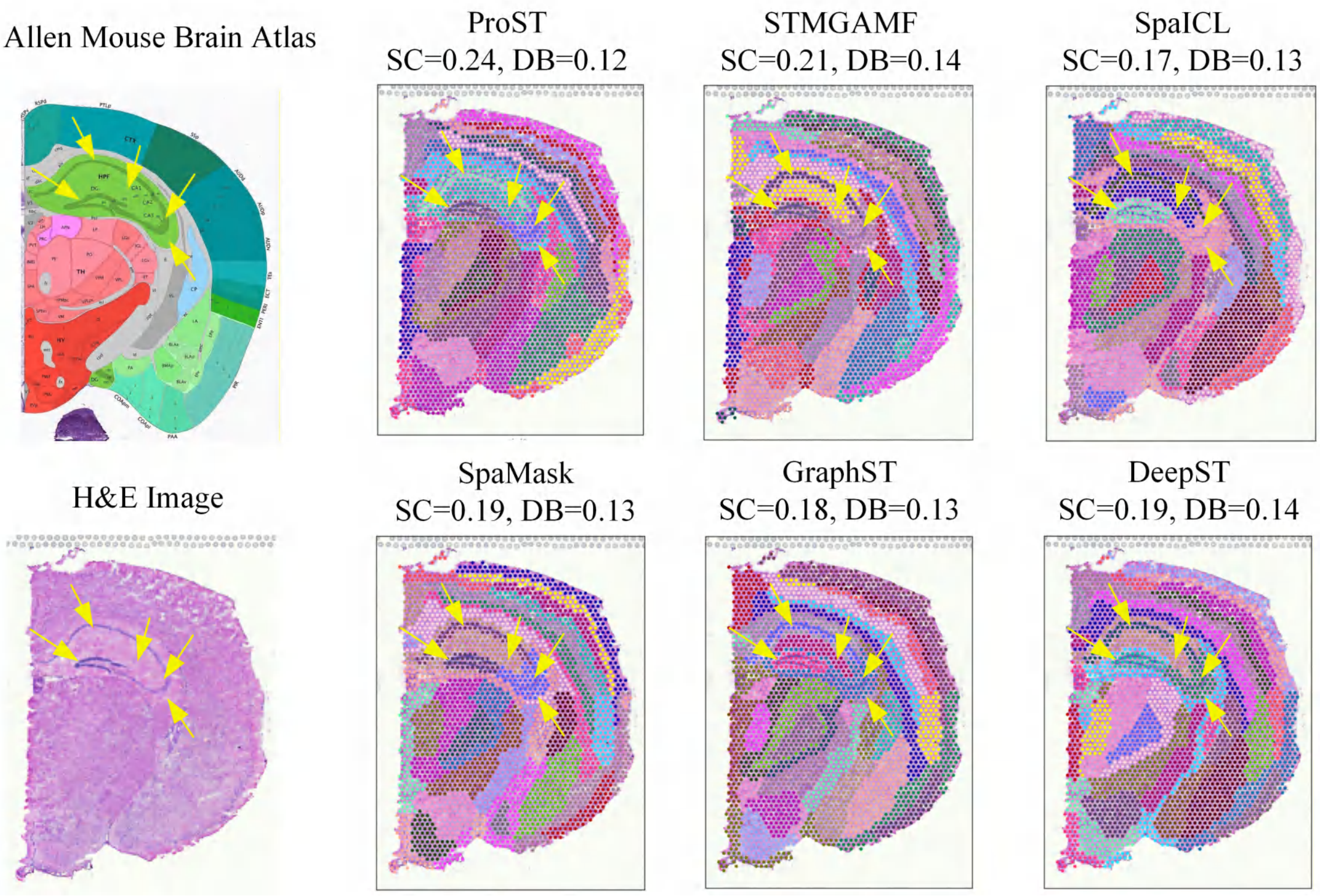

Figure S10. Spatial domain identification on the MBC dataset. In the coronal section, ProST successfully resolves the cerebellar cortex, the dentate gyrus (DG), and the Cornu Ammonis (CA), yielding results highly consistent with the Allen Mouse Brain Atlas. In particular, ProST enables fine-grained characterization of CA sub-regions, including CA1, CA2, and CA3. Although other methods can roughly identify these regions, they are still not sufficiently fine-grained and show inconsistency with the H&E image. This further demonstrates the ability of ProST to precisely resolve complex brain structures in the coronal view.

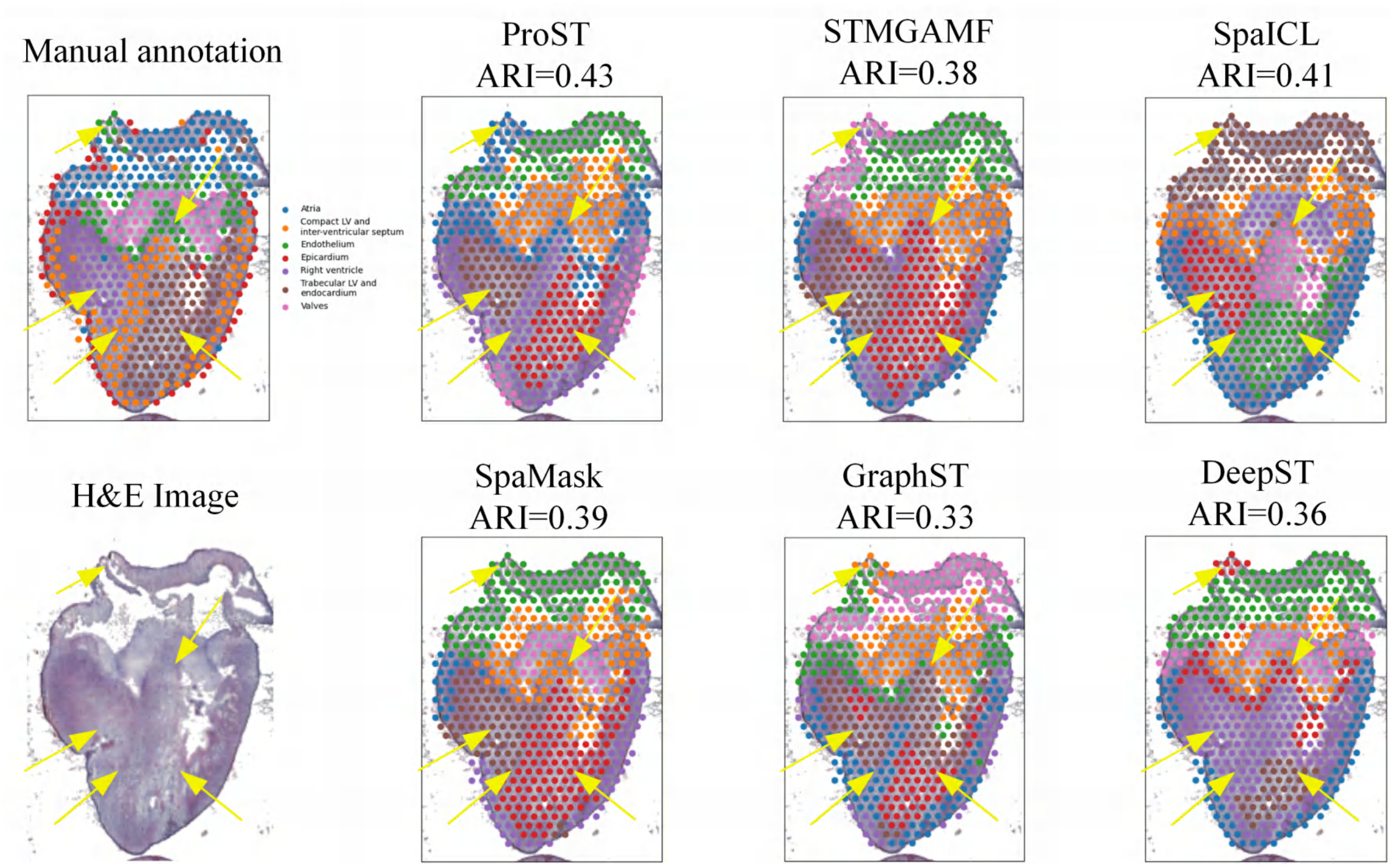

Figure S11. Spatial domain identification on the chicken heart D7 dataset. Compared with the benchmarking methods, ProST achieves the highest ARI, demonstrating its superior ability to resolve cardiac spatial domains in the developing chicken heart. ProST's results are highly consistent with manual annotations, accurately delineating key structures such as the trabecular left ventricle (LV) and endocardium, atria, and right ventricle. In particular, ProST also precisely resolves the compact left ventricle (LV) and the inter-ventricular septum, whereas none of the other compared methods can identify these regions.

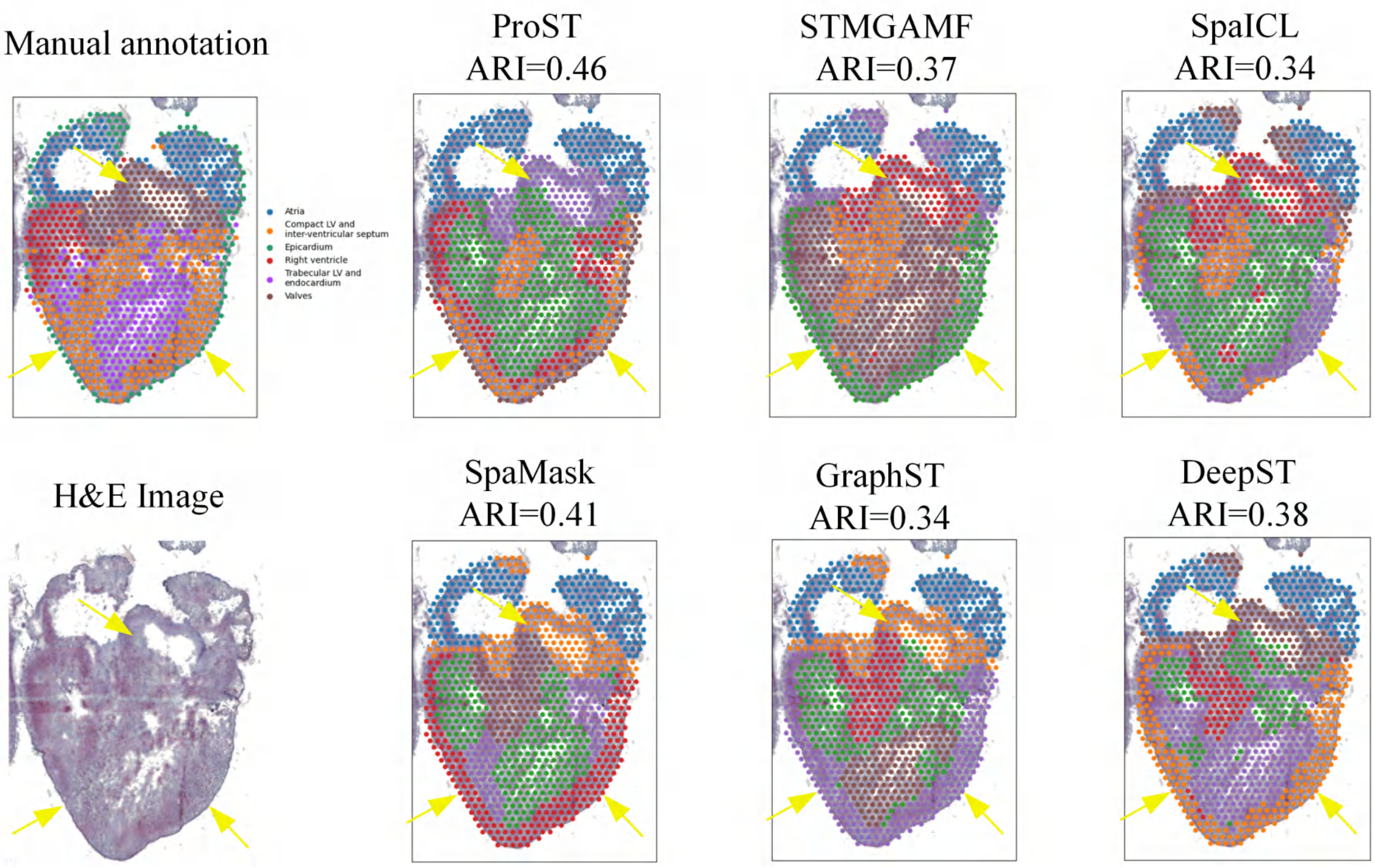

Figure S12. Spatial domain identification on the chicken heart D10 dataset. ProST again achieves the best spatial domain identification performance among all compared methods on the D10 chicken heart section. Its results are highly consistent with manual annotations, accurately delineating key structures including the trabecular left ventricle (LV) and endocardium, atria, right ventricle, as well as the compact left ventricle (LV) and the inter-ventricular septum. In particular, in the epicardium region, only ProST can precisely identify this thin, elongated, fine-grained layer, which completely and continuously surrounds the lower part of the heart. The identification of such a fine structure further demonstrates the excellent capability of ProST and its resistance to over-smoothing.

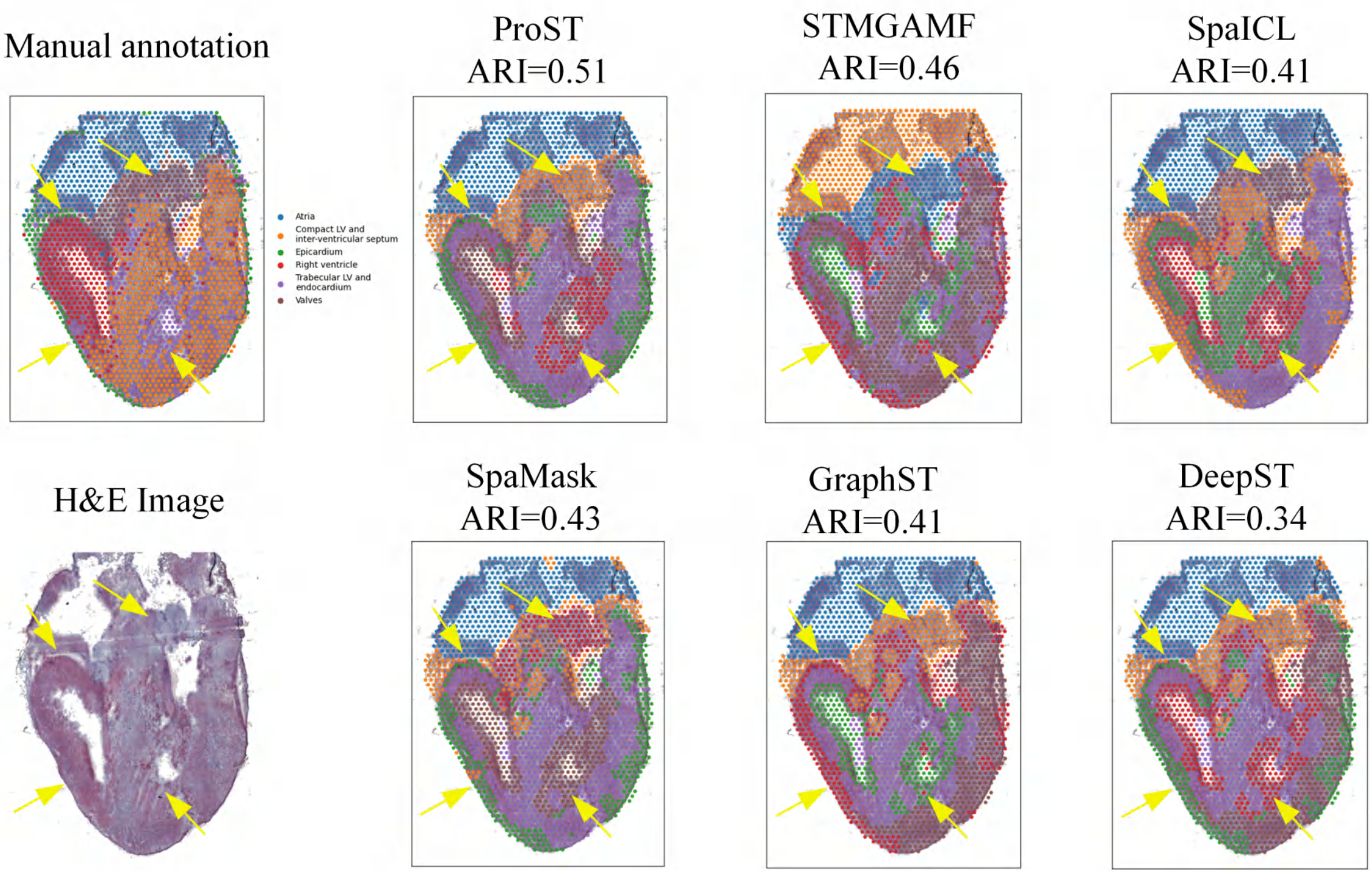

Figure S13. Spatial domain identification on the chicken heart D14 dataset. At D14, myocardial development gradually matures; endocardial cell populations decline and mural cells become significantly enriched, with functional emphasis shifting toward myofibril assembly, striated muscle development, and energy metabolism. Compared with earlier stages, the gene expression landscape at this stage is more complex and spatially heterogeneous, making accurate spatial domain identification more challenging. On this section, ProST again achieves the highest ARI among all compared methods, and its spatial domains are highly consistent with manual annotations. This further indicates that relying solely on gene expression profiles is particularly challenging at this stage, whereas ProST, through its rational multimodal fusion design and with the assistance of histological images, achieves robust spatial domain identification. In particular, ProST clearly separates the right ventricle from the trabecular left ventricle (LV) and endocardium region, whereas other methods tend to merge these two regions into one. Furthermore, ProST identifies a fine hook-shaped epicardium structure above the right ventricle, which is highly consistent with the annotation and continuously and completely surrounds the lower part of the heart. The ability to resolve such subtle structures and maintain clear boundaries further demonstrates the superior capability of ProST.

A

B

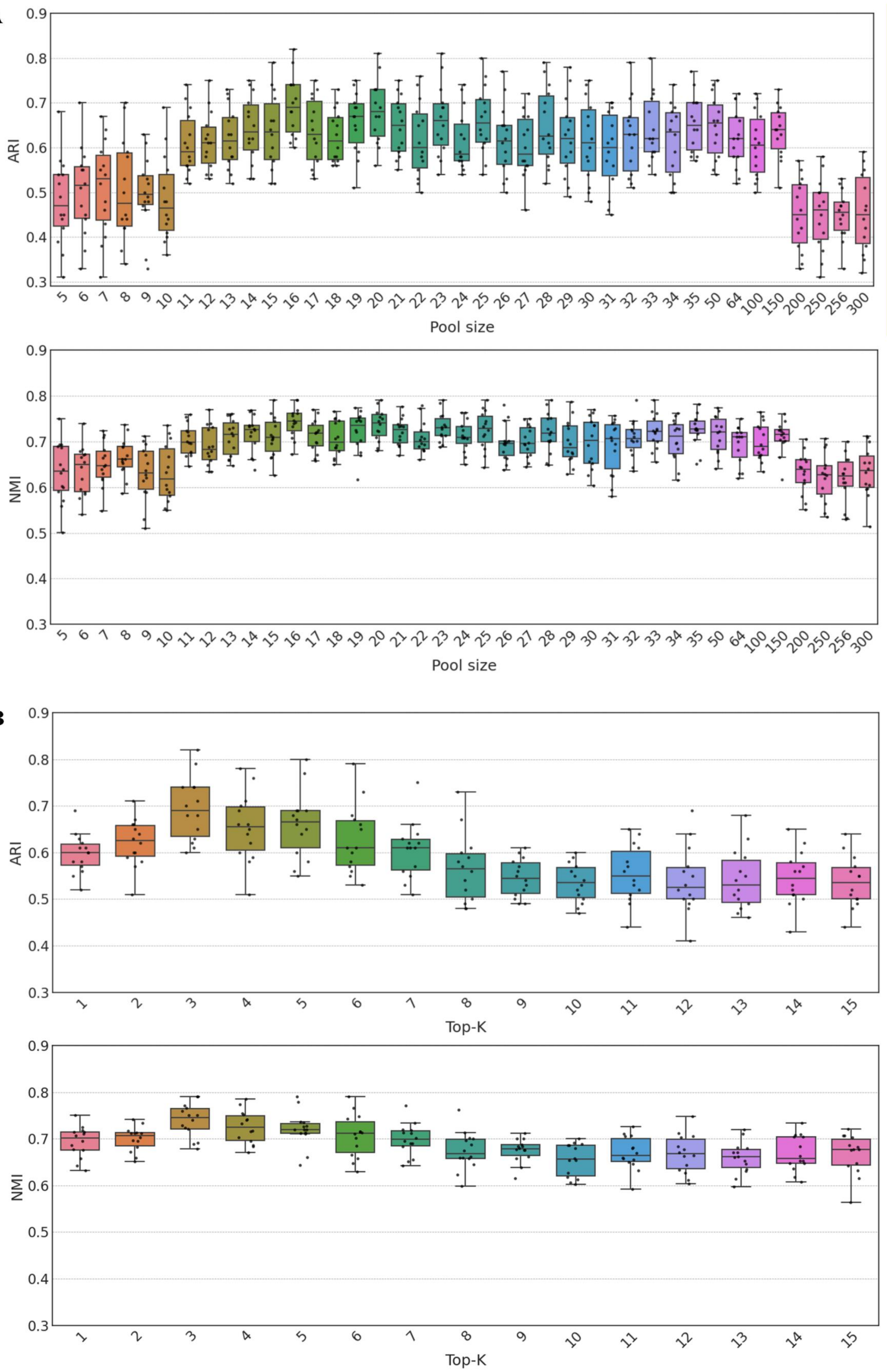

Figure S14. Hyperparameter sensitivity analysis of ProST on the DLPFC, HBC, and MBA datasets: prompt pool size and number of retrieved prompts.

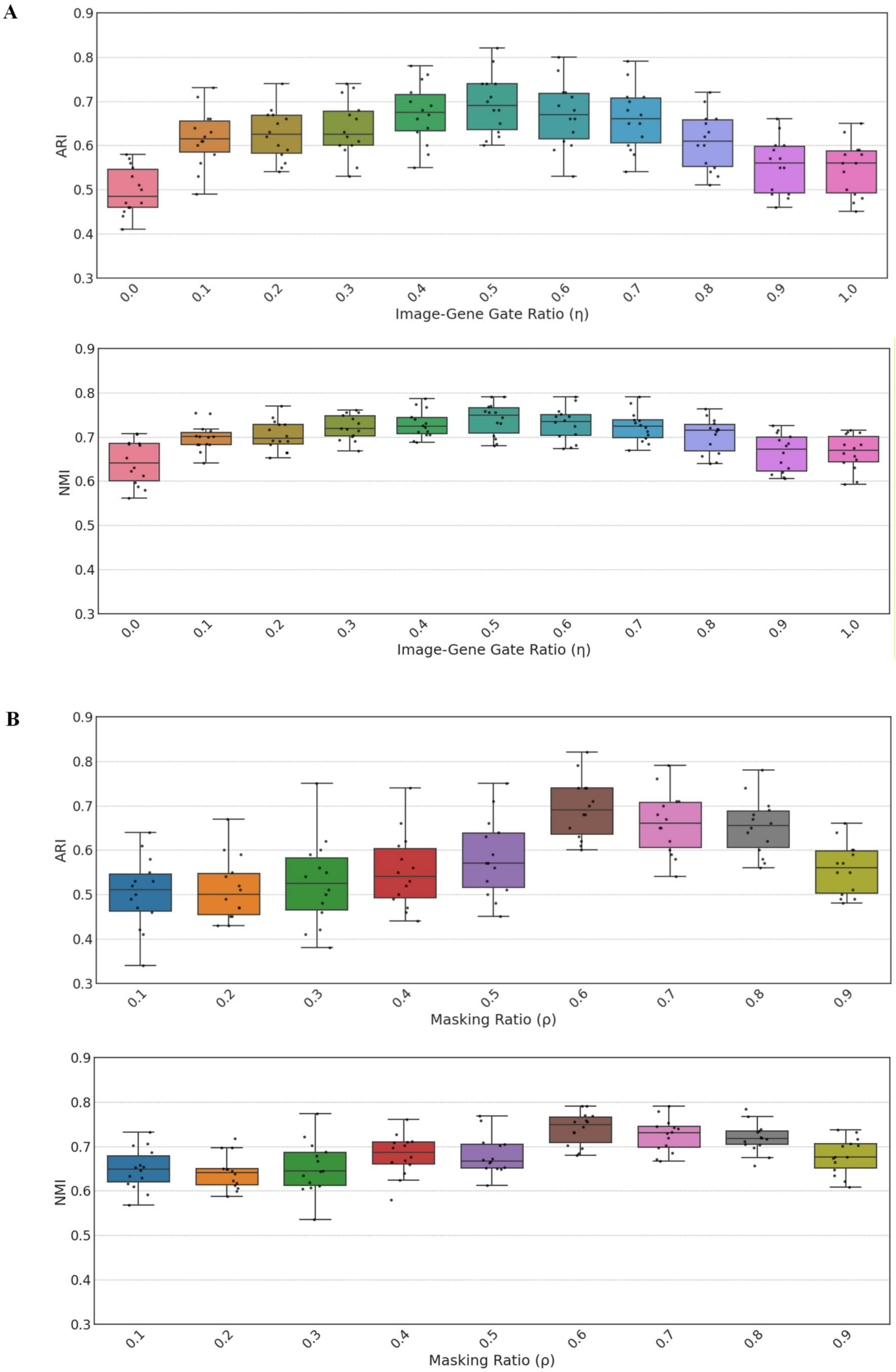

Figure S15. Hyperparameter sensitivity analysis of ProST on the DLPFC, HBC, and MBA datasets: gate ratio and masking ratio.

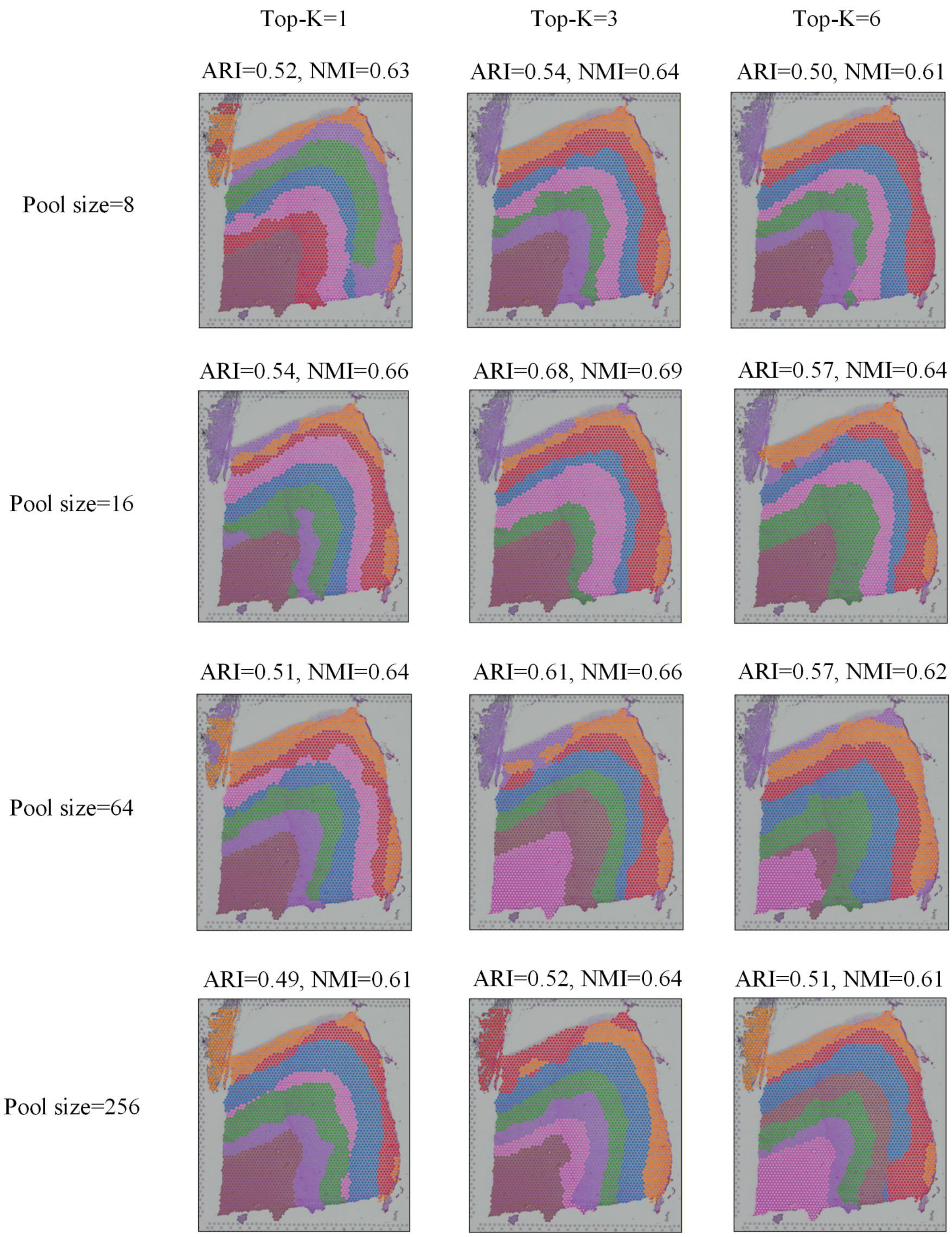

Figure S16. Spatial domain visualization on the DLPFC slice 151675 under different prompt pool sizes (M) and numbers of retrieved prompts (top-K). Each subpanel corresponds to a specific combination of M and K, showing how the identified spatial domains change with these two hyperparameters. The default setting ($M = 16$, $K = 3$) best recovers the annotated cortical layers, whereas smaller or larger values lead to over-fragmentation or over-smoothing of domains. These results visually support the quantitative trends reported in Supplementary Fig. S14A,B.

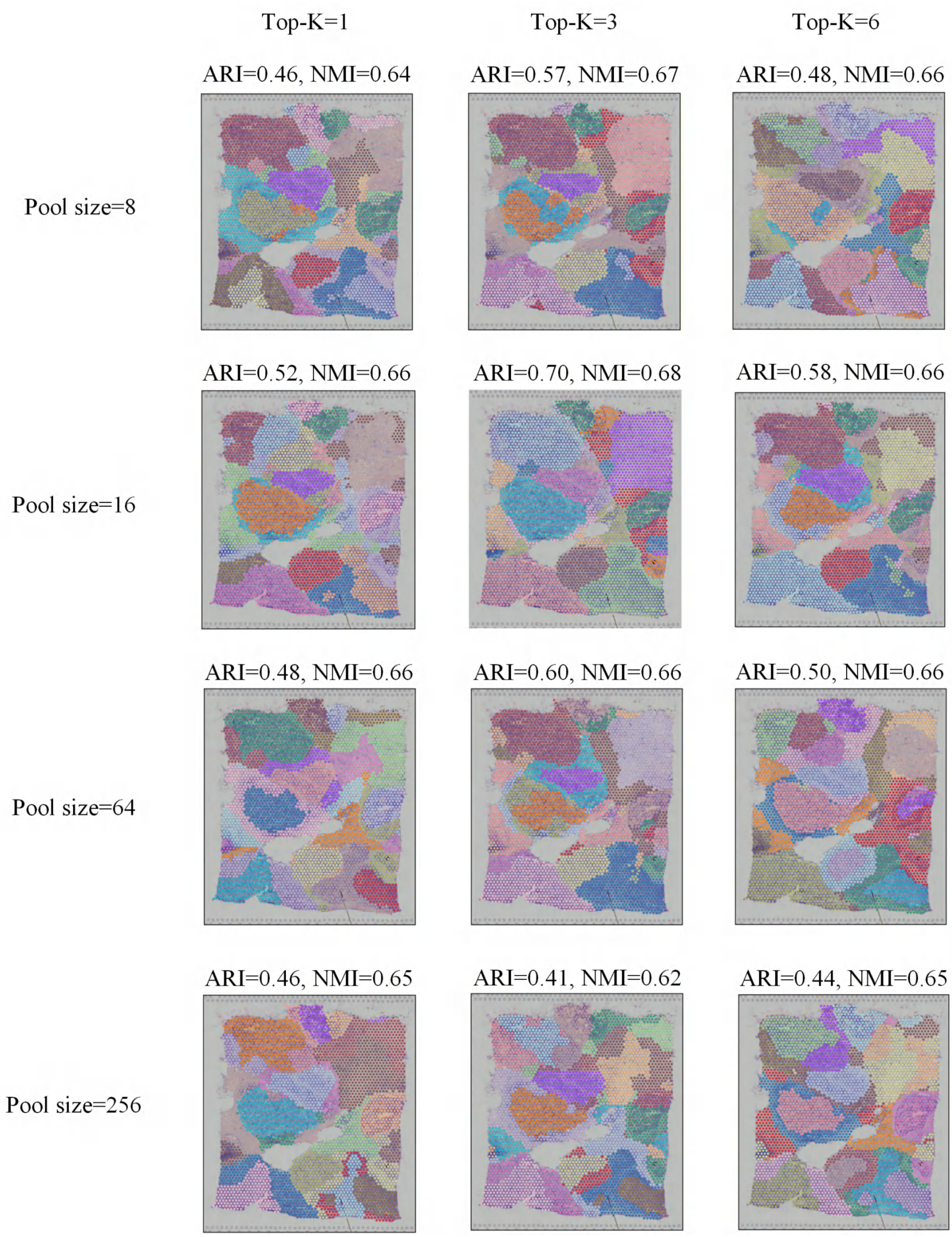

Figure S17. Spatial domain visualization on the HBC dataset under different prompt pool sizes (M) and numbers of retrieved prompts (top-K). Given the highly heterogeneous tumor microenvironment of the HBC section, each subpanel shows how varying M and K affects the delineation of tumor subregions and stromal boundaries. The default configuration ($M = 16$, $K = 3$) yields spatial domains most consistent with the manual annotations, while extreme values introduce spurious boundaries or merge distinct histological compartments. This visualization complements the quantitative analysis in Supplementary Fig. S14A,B.

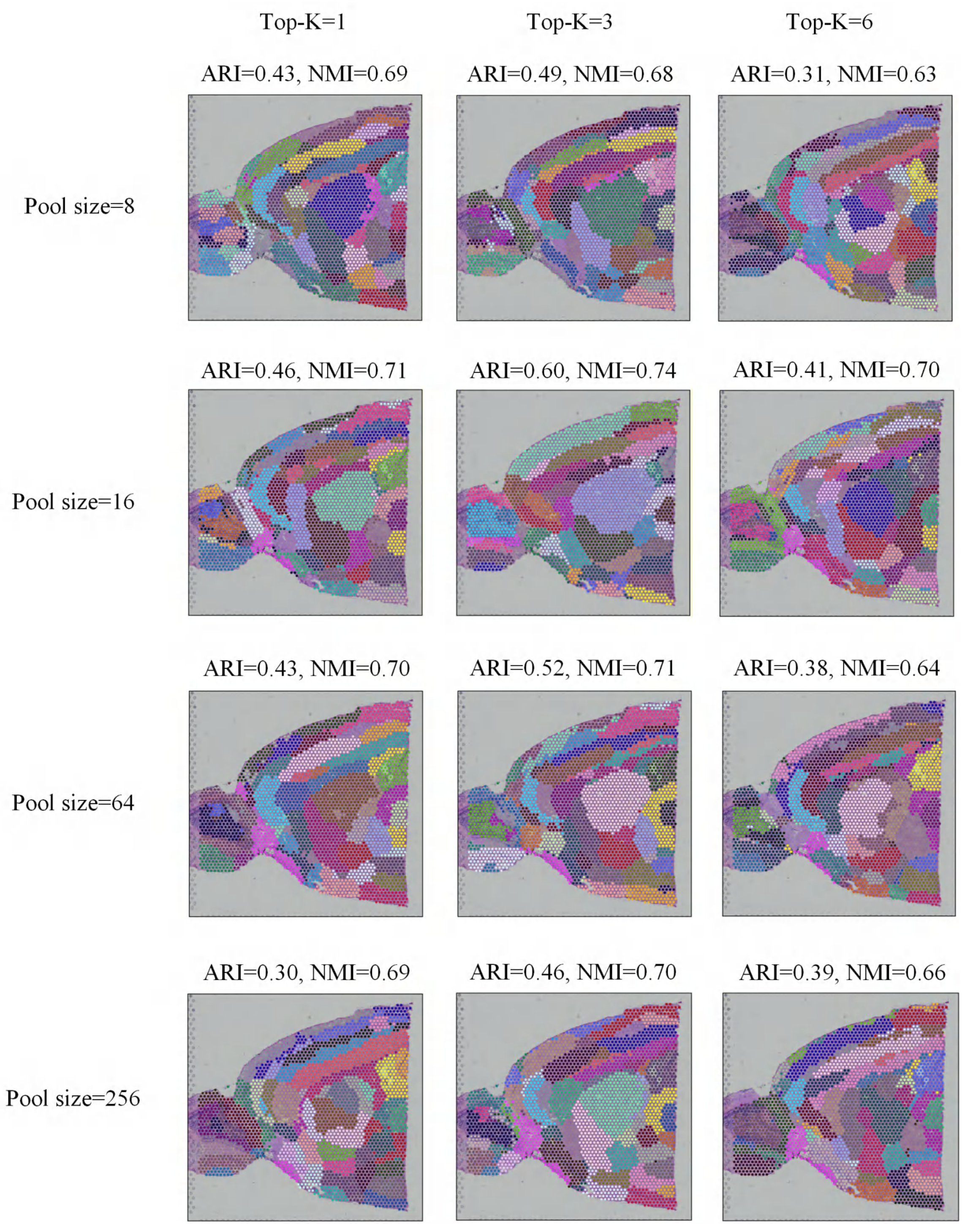

Figure S18. Spatial domain visualization on the MBA dataset under different prompt pool sizes (M) and numbers of retrieved prompts (top-K). The MBA section contains complex and irregular brain structures; the panels illustrate how the choice of M and K influences the separation of fine anatomical regions such as the olfactory bulb and cortical layers. Consistent with the quantitative results, the default settings ($M = 16$, $K = 3$) provide the clearest correspondence to the annotated brain regions, whereas suboptimal values cause domain merging or over-segmentation. These examples reinforce the robustness of the chosen hyperparameters across distinct tissue architectures.

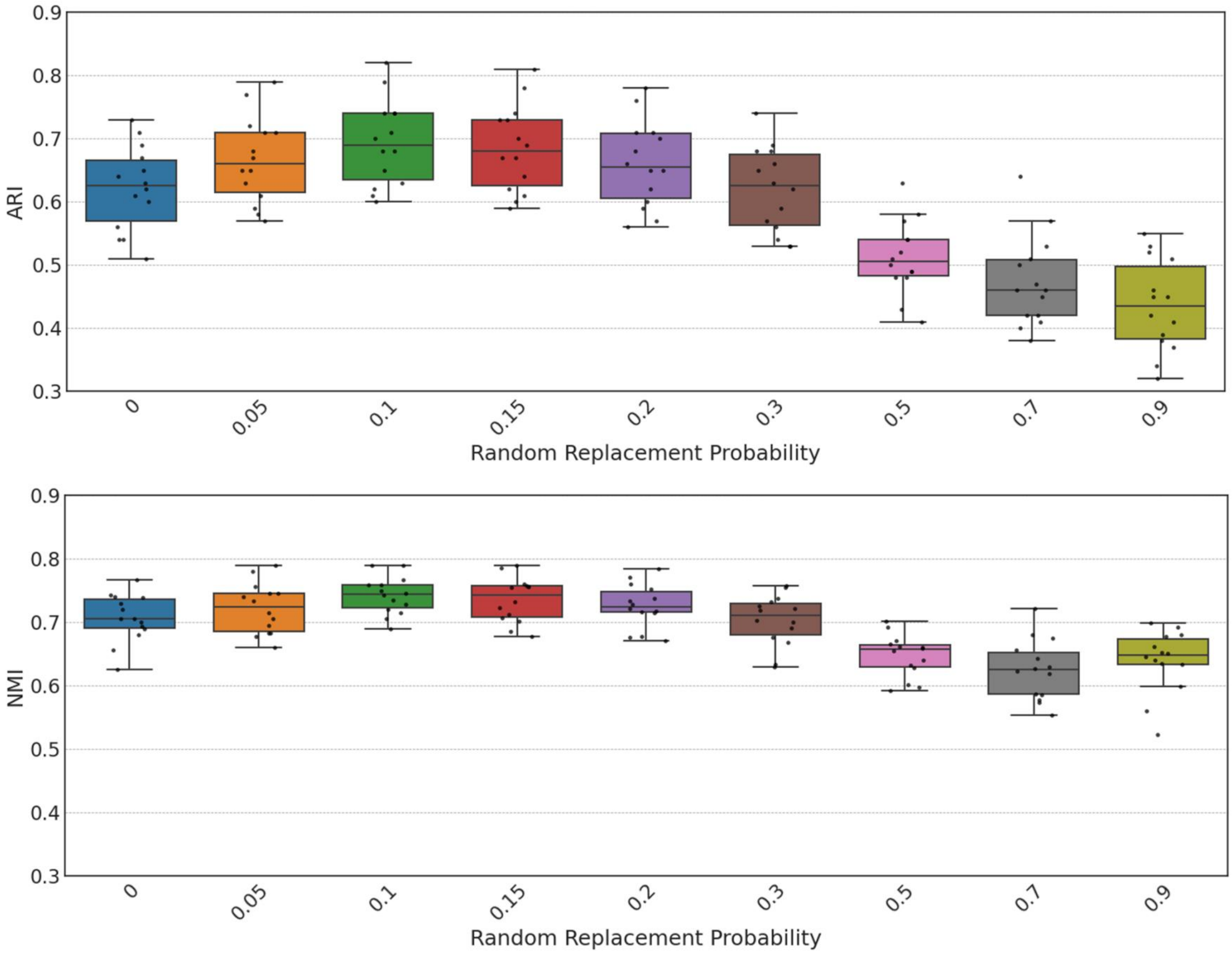

Figure S19. Random Replacement Probability sensitivity analysis.

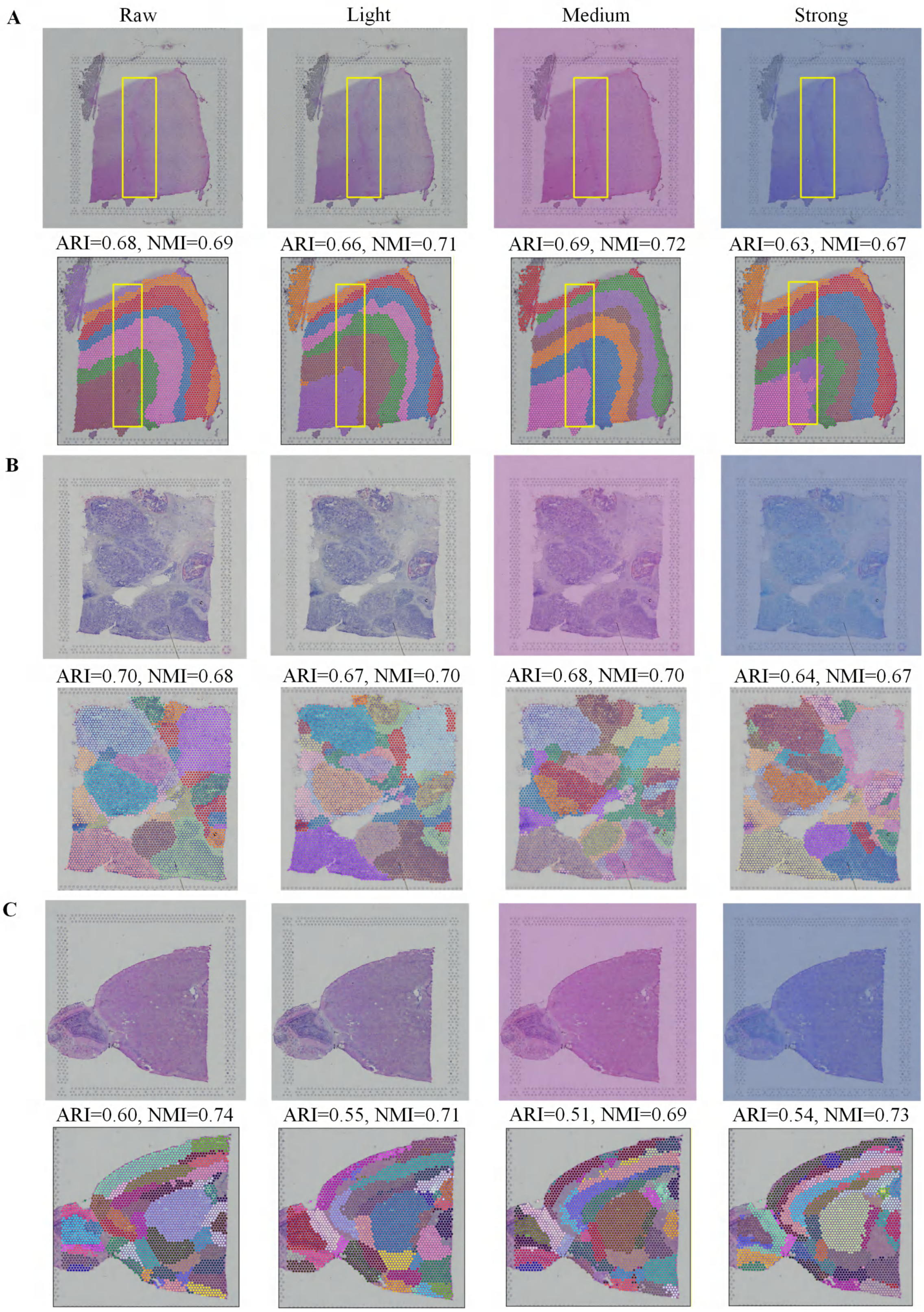

Figure S20. Robustness of ProST to simulated staining variations. Under increasing color jitter, channel scaling/offset, and Gaussian noise on DLPFC, HBC, and MBA, ARI/NMI remained stable except for slight degradation at strong level. ProST consistently excluded the staining artifact (yellow box) and preserved laminar structure in DLPFC 151675.

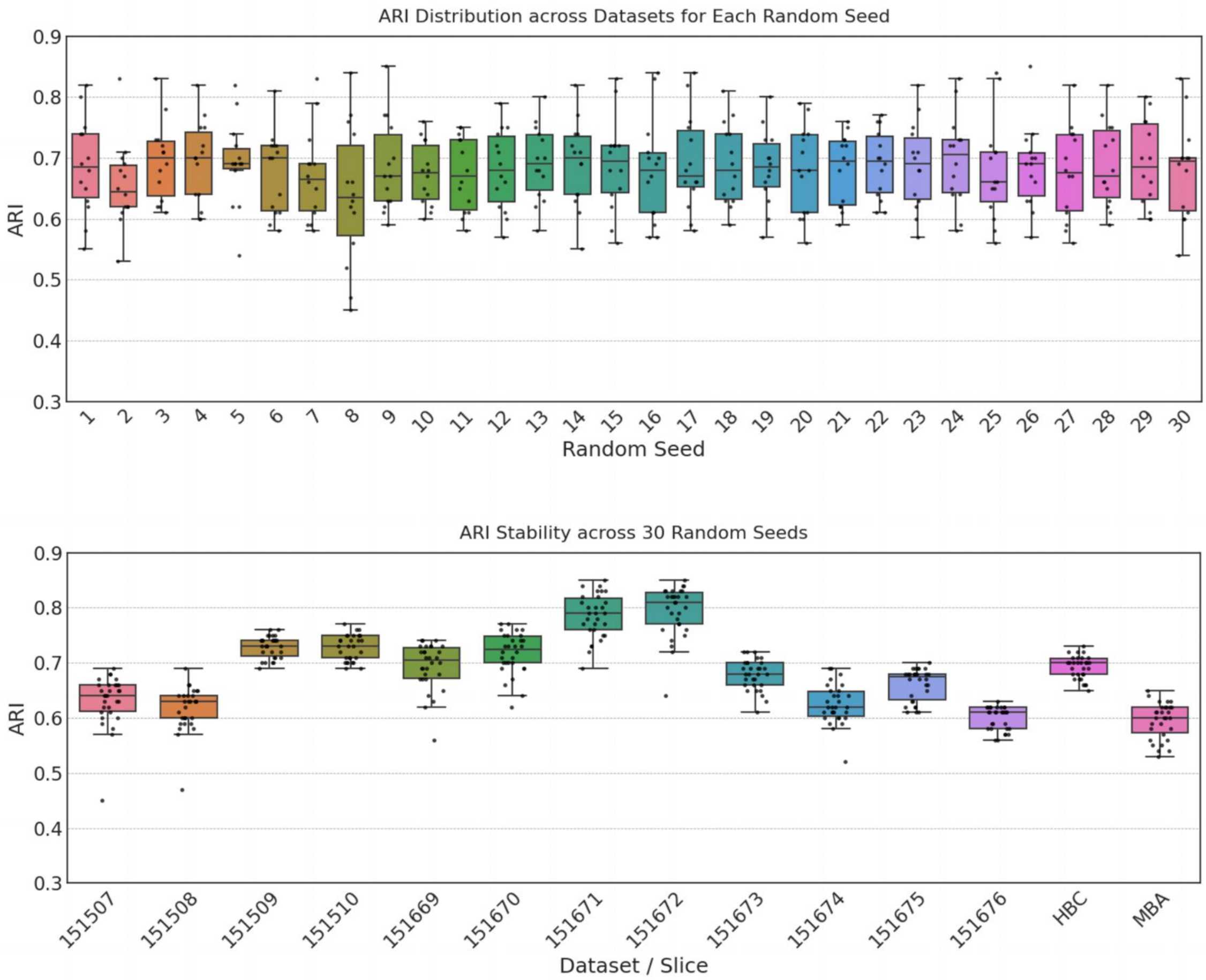

Figure S21. Stability analysis of ProST across 30 random seeds on the DLPFC, HBC, and MBA datasets. For the DLPFC dataset, all 12 tissue slices were evaluated separately, while the HBC and MBA datasets were also included to assess cross-dataset robustness. Each boxplot summarizes the ARI distribution obtained from 30 independent runs with different random seeds for the corresponding dataset or tissue slice. ProST maintained relatively stable ARI distributions across random initializations, indicating that its spatial domain identification performance was not strongly affected by stochastic training variations. Although the absolute ARI values varied across datasets and DLPFC slices due to differences in tissue structure, annotation complexity, and spatial domain difficulty, the within-dataset variations across random seeds remained moderate. These results support the robustness and reproducibility of ProST.

References

[1] Kristen R Maynard, Leonardo Collado-Torres, Lukas M Weber, Cedric Uytingco, Brianna K Barry, Stephen R Williams, Joseph L Catallini, Matthew N Tran, Zachary Besich, Madhavi Tippani, et al. Transcriptome-scale spatial gene expression in the human dorsolateral prefrontal cortex. *Nature Neuroscience*, 24(3):425–436, 2021.

[2] Kornelia Polyak et al. Heterogeneity in breast cancer. *The Journal of clinical investigation*, 121(10):3786–3788, 2011.

[3] Susan M Sunkin, Lydia Ng, Chris Lau, Tim Dolbeare, Terri L Gilbert, Carol L Thompson, Michael Hawrylycz, and Chinh Dang. Allen brain atlas: an integrated spatio-temporal portal for exploring the central nervous system. *Nucleic Acids Research*, 41(D1):D996–D1008, 2012.

[4] Alma Andersson, Ludvig Larsson, Linnea Stenbeck, Fredrik Salmén, Anna Ehinger, Sunny Z Wu, Ghamdan Al-Eryani, Daniel Roden, Alex Swarbrick, Åke Borg, et al. Spatial deconvolution of her2-positive breast cancer delineates tumor-associated cell type interactions. *Nature Communications*, 12(1):6012, 2021.

[5] Madhav Mantri, Gaetano J Scuderi, Roozbeh Abedini-Nassab, Michael FZ Wang, David McKellar, Hao Shi, Benjamin Grodner, Jonathan T Butcher, and Iwijn De Vlaminck. Spatiotemporal single-cell rna sequencing of developing chicken hearts identifies interplay between cellular differentiation and morphogenesis. *Nature Communications*, 12(1):1771, 2021.

[6] Richard J Chen, Tong Ding, Ming Y Lu, Drew F K Williamson, Guillaume Jaume, Andrew H Song, Bowen Chen, Andrew Zhang, Daniel Shao, Muhammad Shaban, et al. Towards a general-purpose foundation model for computational pathology. *Nature Medicine*, 30(3):850–862, 2024.

[7] Ming Y Lu, Bowen Chen, Drew F K Williamson, Richard J Chen, Ivy Liang, Tong Ding, Guillaume Jaume, Igor Odintsov, Andrew Zhang, Long Phi Le, et al. A visual-language foundation model for computational pathology. *Nature Medicine*, 30(3):863–874, 2024.

[8] Kaiming He, Xiangyu Zhang, Shaoqing Ren, and Jian Sun. Deep residual learning for image recognition. In *Proceedings of the IEEE conference on computer vision and pattern recognition*, pages 770–778, 2016.

[9] Christian Szegedy, Vincent Vanhoucke, Sergey Ioffe, Jon Shlens, and Zbigniew Wojna. Rethinking the inception architecture for computer vision. In *Proceedings of the IEEE conference on computer vision and pattern recognition*, pages 2818–2826, 2016.

[10] Jacob Devlin, Ming-Wei Chang, Kenton Lee, and Kristina Toutanova. Bert: Pre-training of deep bidirectional transformers for language understanding. In *Proceedings of the 2019 Conference of the North American Chapter of the Association for Computational Linguistics: Human Language Technologies, Volume 1 (Long and Short Papers)*, pages 4171–4186, 2019.
